# Deployment and validation of the CLL treatment infection model adjoined to an EHR system

**DOI:** 10.1038/s41746-024-01132-6

**Published:** 2024-06-05

**Authors:** Rudi Agius, Anders C. Riis-Jensen, Bettina Wimmer, Caspar da Cunha-Bang, Daniel Dawson Murray, Christian Bjorn Poulsen, Marianne B. Bertelsen, Berit Schwartz, Jens Dilling Lundgren, Henning Langberg, Carsten Utoft Niemann

**Affiliations:** 1 Department of Hematology, Rigshospitalet, Copenhagen University Hospital, Copenhagen, Denmark; 2 SP Sundhedsdata, The Data Unit, Capital Region of Denmark, Copenhagen, Denmark; 3 Center of Excellence for Health, Immunity, and Infections (CHIP), Rigshospitalet, Copenhagen University Hospital, Copenhagen, Denmark; 4 Department of Hematology, Zealand University Hospital, Roskilde, Denmark; 5 Rigshospitalets Innoovationscenter, Copenhagen University Hospital Rigshopsitalet, Copenhagen, Denmark; 6 Department of Clinical Medicine, University of Copenhagen, Copenhagen, Denmark

**Keywords:** Medical research, Chronic lymphocytic leukaemia

## Abstract

Research algorithms are seldom externally validated or integrated into clinical practice, leaving unknown challenges in deployment. In such efforts, one needs to address challenges related to data harmonization, the performance of an algorithm in unforeseen missingness, automation and monitoring of predictions, and legal frameworks. We here describe the deployment of a high-dimensional data-driven decision support model into an EHR and derive practical guidelines informed by this deployment that includes the necessary processes, stakeholders and design requirements for a successful deployment. For this, we describe our deployment of the chronic lymphocytic leukemia (CLL) treatment infection model (CLL-TIM) as a stand-alone platform adjoined to an EPIC-based Danish Electronic Health Record (EHR), with the presentation of personalized predictions in a clinical context. CLL-TIM is an 84-variable data-driven prognostic model utilizing 7-year medical patient records and predicts the 2-year risk composite outcome of infection and/or treatment post-CLL diagnosis. As an independent validation cohort for this deployment, we used a retrospective population-based cohort of patients diagnosed with CLL from 2018 onwards (*n* = 1480). Unexpectedly high levels of missingness for key CLL-TIM variables were exhibited upon deployment. High dimensionality, with the handling of missingness, and predictive confidence were critical design elements that enabled trustworthy predictions and thus serves as a priority for prognostic models seeking deployment in new EHRs. Our setup for deployment, including automation and monitoring into EHR that meets Medical Device Regulations, may be used as step-by-step guidelines for others aiming at designing and deploying research algorithms into clinical practice.

## Introduction

Electronic Health Records (EHR) continue to increase in both size and complexity. EHR may include long-term patient histories of blood tests, previous comorbidities, interventions, medicines, several OMIC data and imaging^1^. In contrast, most research-based models, and the ones implemented into clinical practice, do away with this complexity entirely by creating very simple rule-based models using a handful of variables. The limited number of variables was recently highlighted in a review of CLL prognostic models, where it was found that, on average, only ten variables are used to develop a CLL prognostic model^2^. Some of these variables are dropped during the development process, and the final model may end up using even fewer variables. For example, the gold standard in CLL-IPI^3^ uses five variables to make a prediction. The net effect of not using more variables or adopting better machine-learning algorithms over time has resulted in a flat-lining of performance for CLL prognostic models over the last 20 years^2^. To achieve accurate and reliable prognostic models, solutions for a number of domain-specific challenges that EHR and disease modeling present us with are being developed; including models that handle diverse modalities^4^, high-missingness^5^, data-shifts^6^, underrepresented patient subgroups^7^, privacy limitations^8^ and small patient cohorts^9^. Despite the heterogeneity of these models, none involve low-dimensional rule-based approaches but rather tend towards more complex models. The complexity of these approaches may be a result of high dimensions, the use of long-term patient histories, the fusion of multiple data modalities, and also the use of ensembles or deep-learning architectures. With this in mind, we have no proof-of-concept examples of how complex research algorithms can be safely and successfully automated into clinical practice and the challenges involved in doing so.

**Fig. 1:**
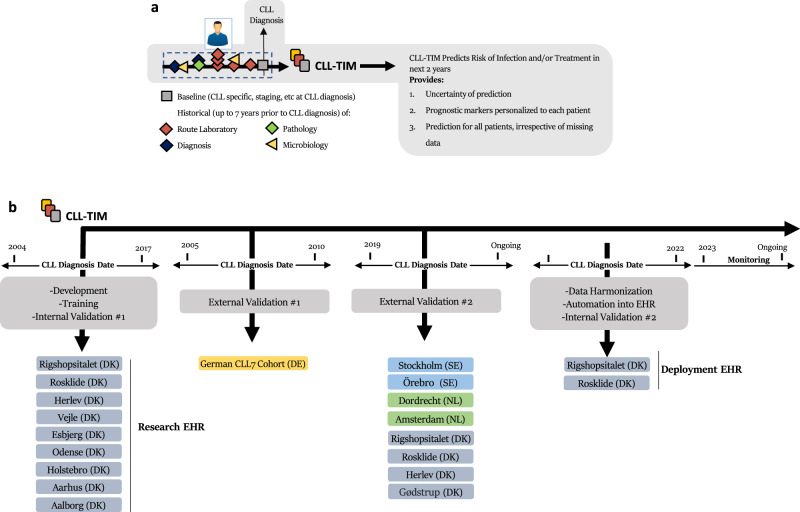
Overview of CLL-TIM’s lifecycle. **a** CLL-TIM was developed as an ensemble model integrating 28 different machine learners trained on data from 4149 patients with CLL as previously described^10^. Patient data for its development and internal validation consisted of patients diagnosed with CLL in Denmark between January 2004 and June 2017. The research database used for development of CLL-TIM (referred here as the research EHR), used all available patient data up until a prediction point of three months post diagnosis for modeling the 2-year risk composite outcome of infection and/or treatment post prediction point. Seven-year historical records of CLL patients were used, and all patient data available was fed into the development of CLL-TIM in a data-driven fashion. Data modalities included laboratory tests, microbiology, diagnosis, and pathology extracted from the PERSIMUNE data warehouse (persimune.org)^27^ and CLL registry-based data as previously described. After feature selection, CLL-TIM consisted of 84 variables spanning 228 features, and on an independent internal test research cohort, CLL-TIM identifies patients with a high-risk (precision of 72% and a recall of 75%) of serious infection and/or CLL treatment within two years from the time of diagnosis. **b** CLL-TIM was validated externally on the CLL7 German cohort and is undergoing further validation in its selection of patients in the PreVent-ACaLL (clinicaltrials.gov: NCT03868722^13^). Patients who, upon their CLL diagnosis, do not need treatment according to iwCLL criteria^31^ but are still deemed as high-risk with high confidence by CLL-TIM are randomized between observation (standard of care) and three months of preemptive venetoclax+acalabrutinib treatment. This is aimed to test whether grade 3-Infection-free, treatment-free survival can be improved. This work describes the deployment of CLL-TIM into the EHR production environment (referred to here as the deployment EHR), the data harmonization process, and the automation of both the data-variable retrieval and the provision of its predictions as views within the deployment EHR. After our deployment, we present CLL-TIM’s performance on a retrospective cohort of patients who were not present during the development of CLL-TIM. We also present our setup for continuous prospective monitoring of CLL-TIM’s performance within the deployment EHR. By design, CLL-TIM is able to provide confidence related to each of its predictions (see Methods—Calculation of CLL-TIM Confidence), explainable predictions unique to each patient, and a prediction for all patients irrespective of their missing data (see Methods—Handling of Missingness in CLL-TIM).

Using CLL as a target disease, we have previously developed the CLL Treatment Infection Model (CLL-TIM) to identify patients newly diagnosed with CLL at high risk of severe infections and/or CLL treatment within two years^10^ (Fig. 1a). As detailed in the primary publication of CLL-TIM, the model was developed based on a clinical unmet need. The major cause of morbidity and mortality for patients with CLL is infection-related^11^, and previous prognostic models did not succeed in identifying newly diagnosed CLL patients who would benefit from early preemptive treatment^12^. Thus, the CLL-TIM model was developed to identify such patients at high risk of severe infections and/or treatment needs within two years of CLL diagnosis and is currently deployed for patient selection in the randomized phase 2 trial PreVent-ACaLL (NCT03868722)^13^. CLL-TIM is an ensemble of 28 algorithms with 84 variables (spanning 228 features) that considers patient data with repeated measurements for up to seven years prior to diagnosis. The complexity was necessary for providing trustworthy predictions, personalized risk factors, and predictions on patients with large amounts of missing data. However, the manual data entry to run the algorithm approaches one hour per patient and is therefore considered prohibitory for the deployment of CLL-TIM into clinical practice as a decision support tool without automation. Therefore, in this work, we aimed to describe and critically assess the deployment of CLL-TIM from a research EHR to a deployment EHR (i.e., production environment). This work comprehensively outlines the transition and automation of CLL-TIM, encompassing external validation, data harmonization, and automation steps. As a final verification, we benchmark our CLL-TIM deployment on a new set of CLL patients including those with key data variables missing. Additionally, our work also details real-time performance monitoring, involvement of stakeholders, and legal frameworks.

## Results

### Data harmonization, automation, stakeholder roles, and legal framework

**Fig. 2:**
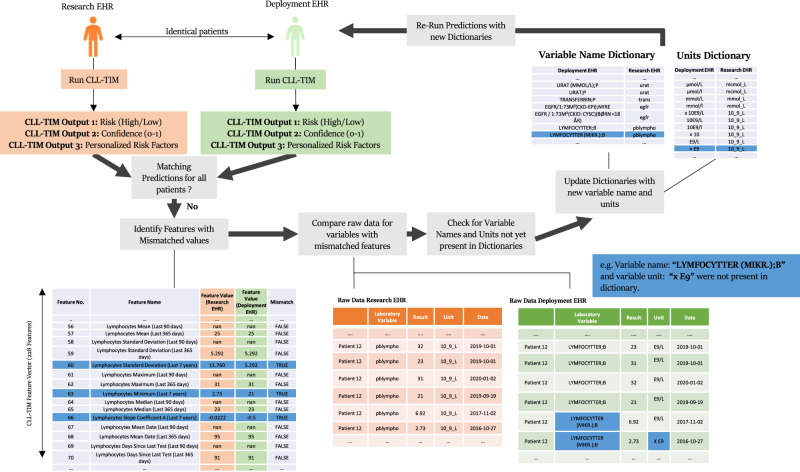
Guiding data harmonization process by matching predictions across cohorts. For each patient, CLL-TIM produces (i) a risk level, which represents the risk of infection and/or treatment with 2-years from its prediction point, (ii) a confidence that ranges from 0 to 1 and represents how sure it is in its prediction (see Methods—Calculation of CLL-TIM Confidence), (iii) a ranked list of features (risk factors) that contribute towards the predicted risk of a given patient. The risk factors and their contribution change for each patient and are, therefore, personalized risk factors. For this harmonization process, we first identified the same patients across both EHRs. The iterative process of data harmonization is complete once CLL-TIM on the same patients across both EHRs achieves the same risk level, confidence, and personalized risk factors. If not, we then identified which of CLL-TIM’s 228 features have unmatched contributions, and from this subset of features, we then extracted which variables were still problematic. Given the list of problematic variables, we could then look into the raw data related to these variables and check for discrepancies. All such discrepancies could be addressed by updating the dictionaries of the variable or the unit mappings accordingly. After updating our dictionaries, we re-ran all patients again to check if further discrepancies were present. Upon achieving a perfect match in CLL-TIM’s three outputs on matched patients across both EHRs, we addressed code optimization issues. Namely, we moved the loading of CLL-TIM’s 28 base learners to occur at the start of the script and for all patient predictions to be run as a single batch prediction. This removed the previous inefficiency of CLL-TIM, where the base learners were re-loaded for each patient prediction.

**Fig. 3:**
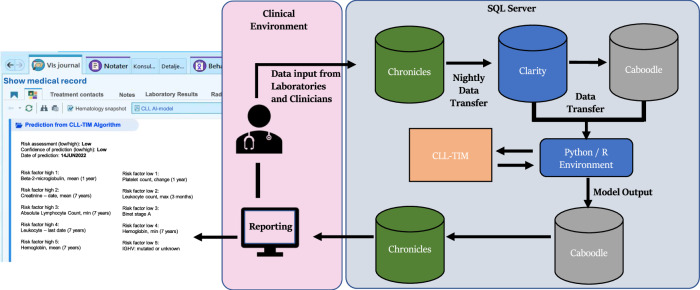
Set up for automated CLL-TIM predictions into deployment EHR. Data is stored in the Chronicles database of the EHR production environment (non-SQL database) upon entering the system from laboratories, diagnostic departments, and clinical personnel. Every night, during an ETL (extract transfer load) process, data is transferred to first the Clarity database and from there into the Caboodle database (both SQL databases) in which data are stored for reporting, data extraction, and monitoring purposes. On the SQL server, within a closed Python environment independently of the EHR system, an area has been dedicated uniquely to CLL-TIM. This closed python environment is managed by the central IT management that ensures identical setups across all hospitals. Data loaded from Chronicles through Clarity to Caboodle databases are passed on to CLL-TIM. Note that before the data enters CLL-TIM, the data harmonization scripts (see Methods) are executed first to ensure that patient data is fed in the right format. The CLL-TIM standalone script is then executed and the predictions are exported to and stored within the Caboodle database. From here, the predictive results are passed back into the Chronicles database, from which direct reporting in the patient chart is presented as a view within the EHR in the patient context, which can be chosen by relevant health professionals to present when accessing EHR for patients with CLL. CLL-TIM’s prediction integrated into EPIC includes the risk of infection and/or treatment for the patient, the confidence in prediction, and the top risk factors pushing towards high-risk or low-risk. The risk factors are personalized for each patient. Both the confidence and personalized risk factors provided may be used by the treating physician to gauge the level of trust one should put in a given patient prediction. In this setup, CLL-TIM is located on the SQL server hosting the databases, thereby minimizing data transfer across different instances. Furthermore, CLL-TIM is containerized in its own environment (Python, R, etc.), ensuring stability when upgrading the environment for any of CLL-TIM’s base algorithms. One important feature of this setup is that the predictions from CLL-TIM and the patient data required to run CLL-TIM are stored on the same database. In this way, our proposed monitoring setup (see Supplementary Methods) need only interact with this database to access both the predictions (to assess performance degradation) and the patient data (to assess data shifts). If found, any such issues may also be similarly reported in the clinical environment interface alongside CLL-TIM’s predictions.

Data harmonization was achieved through the development of mapping dictionaries, which aligned variable nomenclature and units with the specific requirements of CLL-TIM (See Fig. 2 and Methods). This harmonization process was validated by comparing the results of CLL-TIM on the research EHR and the deployment of EHR on an identical set of patients. This included comparing the predicted risk, confidence, and personalized risk factors provided by CLL-TIM. We then setup the importation and exportation of data from and to CLL-TIM’s standalone script in an automated fashion (See Fig. 3 and Methods). Our setup for automation enforces that the input (patient data) and output (predictions) of CLL-TIM, were easily accessible. This enables us to both monitor the performance (using the outputs) and anticipate data shifts present (using the inputs) (See Supplementary Fig. 1 and Methods). Collaboration between an interdisciplinary team was necessary to integrate CLL-TIM’s results into the deployment EHR while also considering all clinical, technical, and regulatory aspects (See Table 1). A Medical Device Regulation (MDR) assessment was performed, and before making available CLL-TIM’s predictions on the visual interface of the deployment EHR, several aspects of CLL-TIM’s benchmarking and validation were also assessed by Hematological Clinical Healthcare Council (see Methods).

**Table 1:** Stakeholder roles

Stakeholder	Role
Researchers	•Defining the clinical scope and impact of the CLL-TIM. •Development of CLL-TIM.
Legal Department	•Assessment of legal status and approval of decision support tool according to MDR regulation and/or CE labeling
Data analysts	•Deploying the CLL-TIM on the deployment EHR system. •Mapping data variables from the research project to data variables in the deployment EHR system. •Setting up automatic execution of CLL-TIM, the data lineage for the input features, and validating the CLL-TIM’s performance on the database.
IT architects	•Defining the implementation pathway of the CLL-TIM.
User interface developers	•Building the user interface report for presenting the CLL-TIM’s output.
Clinical Healthcare council	•Determine how the CLL-TIM’s output should be presented for the clinicians. •Accepting that CLL-TIM output is shown to all clinicians within the relevant clinical area.

Researchers defined the research project and the scope of the CLL-TIM algorithm, including its clinical impact and limitations, and developed the algorithm. Data analysts, IT architects, and User interface developers are all situated within the deployment EHR organization. These are responsible for the implementation of the CLL-TIM algorithm on the deployment EHR system, building the user-interface reporting tool, and designing the continuous monitoring of its performance; the healthcare council has the clinical responsibility that CLL-TIM is widely shown to clinicians in the correct context and representation and within CLL-TIM’s impact area; the business owners prioritize which models should have the main priority in being deployed in the deployment EHR system.

### Comparison of research and deployment EHR cohorts

**Fig. 4:**
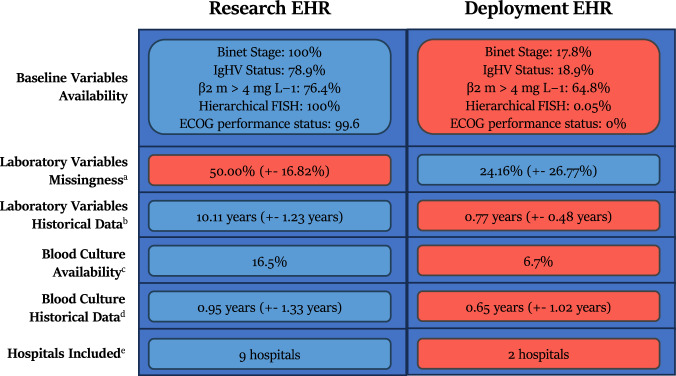
Comparison of missingness in research EHR and deployment EHR data. Research EHR data is patient data that was used for the development, training, and internal validation of CLL-TIM (Patients with CLL diagnosis prior to 2018). Deployment EHR is the patient data that was used to test CLL-TIM’s generalization performance upon its deployment (Patients with CLL diagnosis post-2019). Baseline characteristics are given in Table 2. EHR patient data considered is only up until CLL-TIM’s prediction point, i.e., 3 months post CLL diagnosis. As per our previous findings when performing missingness simulations during CLL-TIM’s development, the blue color indicates what is generally advantageous to have better predictions, and red indicates what is disadvantageous. The missingness of the baseline variables in the deployment EHR was due to them not being available in a structured format, even though they were present in the medical record. A laboratory variable missingness was calculated using the 33 laboratory variables used by CLL-TIM. Namely it shows the mean percentage of laboratory tests each patient has no data for. **b** Historical data was calculated by first considering, for each patient and for each laboratory test, what was the earliest test available. For each laboratory test, we took the mean overall patients, and our final number is the mean overall laboratory tests. This gives an idea of how far back laboratory data is available. Availability and historical data for each laboratory test may be found in Supplementary Fig. 2. **c** Blood culture availability is the rate of patients with at least one blood culture event. **d** Blood culture historical data is the mean number of years a blood culture event took place before the prediction point. Distribution of blood culture events prior to prediction points may be found in Supplementary Fig. 2. **e** CLL-TIM was developed and trained on patient data from Rigshospitalet, Roskilde, Herlev, Vejle, Esbjerg, Odense, Holstebro, Aarhus and Aalborg in Denmark. The deployment cohort includes patient data from Rigshospitalet and Roskilde. These two hospitals formed 23% of the patient population in the research EHR. β2-M Beta-2 microglobulin, IGHV the immunoglobulin heavy chain gene, FISH DNA fluorescence in situ hybridization, ECOG Eastern cooperative oncology group.

CLL-TIM was originally developed, trained, and tested on a research EHR (*n* = 3720) of patients diagnosed with CLL prior to 2018 (Fig. 1). For further validation after deployment, we here tested CLL-TIM on an independent cohort of CLL patients diagnosed post-2018, from the deployment EHR (*n* = 1099) (see Table 2 for baseline characteristics and Supplementary Fig. 1 for Consort Diagram). We here highlight the key data differences exhibited by the research and deployment EHRs (Fig. 4). In the research EHR, within 2 years from the prediction point (set at 3 months post CLL diagnosis), 14.9% of patients had treatment as a first event, 18.1% of patients had an infection as a first event, and 33% patients had the composite event of treatment of infection. For the deployment of EHR, the respective rates were 17.5%, 23.2%, and 40.6% (Supplementary Fig. 1). The research EHR spanned patient data from nine hospitals across Denmark. Deployment EHR included only two of these nine hospitals—Rigshopsitalet and Roskilde. These two hospitals initially encompassed 23% of the training population of CLL-TIM. Patients in the research and deployment EHR showed similar baseline characteristics (Table 2), with the exception of the deployment EHR having a higher percentage of patients (76.4% vs 70.1%) above the age of 65. The research and deployment EHRs, however, showed marked differences in missingness (Fig. 4). For the baseline variables of CLL-TIM i.e., those taken around the time of diagnosis, missingness for the research vs. the deployment EHR were as follows: Binet Staging (0% vs 72.2%), β2 microglobulin (23.6% vs 35.8%), IGHV mutation status (21.1% vs 91.1%), Hierarchical FISH (0% vs 99.5%) and ECOG performance status (0.4% vs 100%). For the deployment of EHR, the high missingness of the aforementioned variables was due to them not being available in a structured format, even though they were mostly available in the medical records. For the research EHR, these variables were manually entered into a structured format. In the research EHR, the percentage of patients with a missing laboratory test was double that of the deployment EHR. This is because the research EHR spanned a wider range of hospitals, most of which had less data in structured format. The deployment EHR had, on average, less than a year of laboratory historical data (vs. ten years for the research cohort). The deployment of EHR had a lower incidence of patients with blood culture events. In the deployment of EHR, blood cultures were generally only available from 2017 and thus also exhibited a shorter historical availability. Diagnosis, pathology, and microbiology data were not extracted for the current deployment of CLL-TIM as their contribution to CLL-TIM’s predictions was found to be minimal during missingness simulations^10^. Future updates of the CLL-TIM deployment will also add these variables.

**Table 2:** Comparative baseline characteristics of research and deployment EHR patient cohorts

Variable	Level	Research EHR (*n* = 3720)	Deployment EHR (*n* = 1480)
Age (years)	<65 years	1114 (29.9)	349 (23.6)
	≥65 years	2606 (70.1)	1131 (76.4)
Sex	Female	1480 (39.8)	596 (40.3)
	Male	2240 (60.2)	884 (59.7)
Binet stage	A	3177 (85.4)	363 (88.1*)
	B	443 (11.9)	26 (6.5*)
	C	100 (2.7)	23 (5.6*)
β2-M > 4 mg L^−1^	No	2498 (88.0)	806 (84.8*)
	Yes	342 (12.0)	144 (15.2*)
IGHV status	mutated	2063 (70.3)	92 (70.2*)
	unmutated	873 (29.7)	39 (29.8*)

Research EHR refers to the baseline characteristics for *n* = 3720 patients diagnosed with CLL prior to 2018 and used in the development, training and internal validation of CLL-TIM. Deployment EHR refers to the baseline characteristics for the *n* = 1480 patients diagnosed with CLL post-2018 that were used for prospective benchmarking of CLL-TIM after its deployment. Note that the deployment EHR exhibited high missingness for Binet Stage (72% missing), IGHV (91%). Further details and comparison of missingness to the research EHR for key CLL-TIM variables are presented separately in Fig. 4. *IGHV* the immunoglobulin heavy chain gene, *β2-M* β2 microglobulin. *Percentages for these groups are calculated with respect to patients with available data for the given variable and not on the overall population.

### Benchmarking deployment of CLL-TIM on an independent cohort of patients

**Fig. 5:**
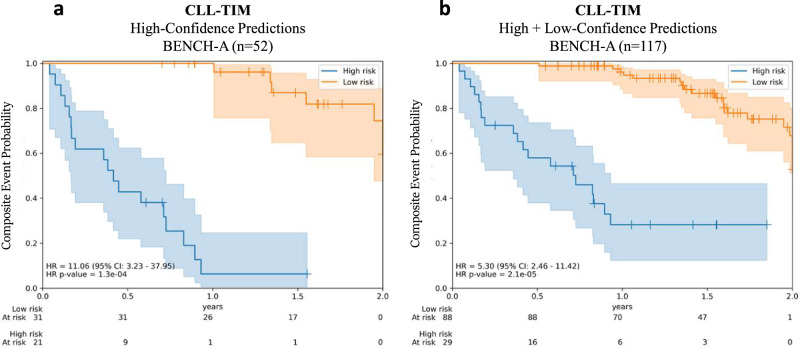
Kaplan–Meier curves for composite outcome predictions of CLL-TIM on deployment EHR. Kaplan–Meier graphs of infection-free, CLL treatment-free survival for CLL-TIM’s predictions. Patients predicted as high-risk is the blue curve with 95% confidence intervals) and the low-risk group is the yellow curve with 95% confidence intervals. p-value is by log-rank test. **a** All predictions shown are for high-confidence predictions. For each risk prediction, CLL-TIM provides confidence in its prediction that is given without knowledge of the ground truth. The threshold for a high-confidence prediction was 0.58, and any patients with a confidence level higher than 0.58 were considered to be high-risk with high confidence. For low-risk predictions, the high-confidence threshold was set at 0.28, and any patients with a confidence level less than 0.28 were considered to be low-risk with high confidence. Thresholds were derived from the original CLL-TIM publication^10^ and unchanged for this work. Derivation of these confidences and the thresholds is described in Methods. **b** Includes also patients in which CLL-TIM had low-confidence in its prediction. For an equitable comparison to CLL-TIM’s predictions on the research EHR, the results here are for the subset of patients with available Binet Stage and IGHV status but for missingness in any other variables.

On the research EHR, on a set of patients separate from those that CLL-TIM was trained on, CLL-TIM achieved Matthew’s correlation coefficient (MCC) of 0.56 (95% CI: 0.42–0.70) (*n* = 145) on the subset of patients with no missing CLL-IPI variables (Binet Staging, IGHV mutational status, β2 microglobulin, del17p/TP53 mutation) and on which CLL-TIM showed high-confidence in its prediction^10^. Note that this initial benchmarking allowed missingness for any other variables. For an equitable comparison to test CLL-TIM’s generalization from the research EHR to the deployment EHR, we wanted to test CLL-TIM’s predictions in the deployment EHR on the subset of patients with non-missing CLL-IPI variables. Due to the limited availability of these variables (Fig. 4), we restricted it to non-missing Binet Staging and IGHV only. All other variables were not controlled for missingness. On this subset of the patients from the deployment EHR (*n* = 31, Table 3—Bench A), the high-confidence predictions of CLL-TIM achieve an MCC of 0.715 (95% CI: 0.712–0.719) with a precision of 0.942 (95% CI: 0.941–0.944) vs. 0.72 (95% CI: 0.63–0.90) and a recall of 0.852 (95% CI: 0.849–0.854) vs. 0.754 (95% CI: 0.65–0.86) for CLL-TIM on the research EHR respectively. Survival analysis (Fig. 5a) on the high-confidence predictions of CLL-TIM on the deployment EHR exhibited a hazard ratio (HR) of 11·06 (95% CI: 3.23–37.95) vs. 7.23 (95% CI: 7.19–7.52) for CLL-TIM on the Research EHR.

**Table 3:** Comparative benchmarking of high-confidence predictions of CLL-TIM on deployment EHR vs research EHR

		Research EHR (*n* = 145)	Deployment EHR BENCH-A (*n* = 31)	Deployment EHR BENCH-B (*n* = 276)	Deployment EHR BENCH-C (*n* = 233)
Missingness control		CLL-IPI variables non-missing	Binet Stage and IGHV non-missing (two of the CLL-IPI variables)	As-is missingness	As-is missingness
% Availability	Binet stage	100%	100%	28%	28%
	IGHV	100%	100%	9%	9%
	β2M	100%	64%	64%	64%
	ECOG status	100%	0%	0%	0%
	FISH status	100%	0.05%	0.05%	0.05%
	Other CLL-TIM Variables	Varied	Varied	Varied	Varied
Metrics	PR-AUC	0.778 (0.691–0.859)	0.979 (0.978–0.979)*	0.598 (0.597–0.600)*	0.573 (0.571-0.575)*
	MCC	0.558 (0.418–0.697)	0.715 (0.712–0.719)*	0.500 (0.499–0.501)	0.518 (0.516-0.520)
	Precision	0.719 (0.631–0.895)	0.942 (0.941–0.944)*	0.582 (0.581–0.582)*	0.618 (0.617-0.619)*
	Recall	0.754 (0.649–0.86)	0.852 (0.849–0.854)	0.786 (0.785–0.787)	0.679 (0.677-0.681)
	TP	43	17.884 (17.838–17.930)	66.828 (66.725–66.931)	38.691 (38.593–38.789)
	FP	17	1.137 (1.110–1.165)	48.324 (48.156–48.491)	24.136 (24.008–24.264)
	TN	71	8.863 (8.835–8.890)	142.676 (142.509–142.844)	141.864 (141.736–141.992)
	FN	14	3.116 (3.070–3.162)	18.172 (18.069–18.275)	18.309 (18.211–18.407)

Predictions for Research EHR were assessed on an internal research benchmark during the development of CLL-TIM. Namely, a subset of patients in the test cohort with full CLL-IPI and a full two-year follow-up. All patients in this set were diagnosed with CLL prior to 2018. For deployment of EHR, patients were diagnosed with CLL post 2018 and, therefore, were not part of the development of CLL-TIM. The prediction point was at three months post-CLL diagnosis, and the binary outcome predicted was whether a patient had an infection or needed CLL treatment within 2 years from the prediction point. For this classification analysis, patients with no event and a follow-up of less than 2 years were removed from the analysis (for analysis with these patients included through censoring, please see Kaplan-Meier curves in Fig. 5a). All predictions shown are for high-confidence predictions. Results for the Research EHR are taken from the original publication of CLL-TIM and calculated on patients for which CLL-IPI variables were completely available. Bench-A—for an equitable comparison we present results for CLL-TIM on the deployment EHR, on the subset of patients with available Binet Stage and IGHV status. Bench-B—shows performance on all patients with any missing data. Bench-C—identical benchmark to Bench-B, except that we tested the use of calibration. Namely, we increased the threshold of what is considered to be high-risk with high confidence from 0.58 (derived from the original CLL-TIM publication) to 0.65. PR-AUC area under the precision-recall curve, MCC Matthew’s correlation coefficient, TP, FP, TN, FN are true-positive, false-positive, true-negative, false-negative predictions for the 2-year outcome, IGHV the immunoglobulin heavy chain gene, β2M β2 microglobulin, FISH DNA fluorescence in situ hybridization, ECOG Eastern cooperative oncology group. Results are for 5000 bootstrapped patient cohorts where sampling was performed with replacement and stratified to preserve original high-risk to low-risk patient ratios within each bootstrapped dataset. We then generated 95% confidence intervals (shown in brackets) for each metric over the bootstrapped datasets. * Indicates that the difference between the measurements (Research EHR vs Bench A, B, or C) is statistically significant as 95% confidence intervals do not overlap. The comparison was limited to PR-AUC, MCC, Precision, and Recall.

### Effect of missingness on CLL-TIM’s predictions on the deployment of EHR

**Fig. 6:**
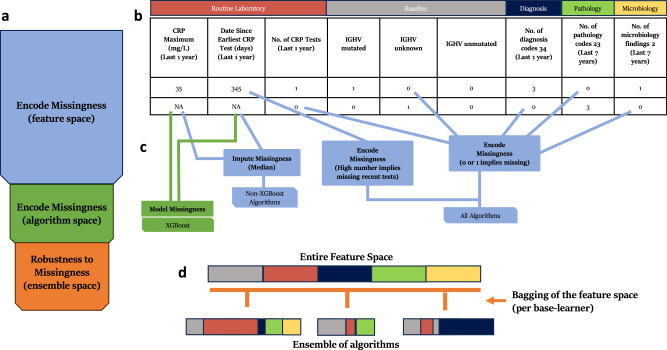
The 3 layers of handling missing data in CLL-TIM. **a** CLL-TIM handles missingness using 3 different layers. Namely, (i) on the feature level using missingness representations, (ii) on the algorithm level by using algorithms that accept missingness, and (iii) on the ensemble level by using design principles that make it robust to missingness and noise (see Methods—Handling of Missingness in CLL-TIM). **b** Shows how CLL-TIM handles missingness on the feature level. For routine laboratory variables, the features that count the number of a given test can indicate to the algorithm that a test is missing when its value is 0. For baseline variables, since they are categorical, one-hot-encoding was used to create missingness indicator features. For example, ‘IGHV unknown’ indicates to the algorithm that the variable is missing. Similarly, for Diagnosis, Pathology, and Microbiology, a count of 0 implies the respective feature is missing. These missingness indicators enable the subsequent algorithms to include the missingness as part of their decision-making. For example, the algorithm might employ proxy features to model patients with missing IGHV status. Sometimes, missingness is time-dependent. For example, a laboratory test might be missing in the last year but not in the last 4 years. So, another type of feature that represents missingness is one that counts the number of days from a test. High values for this feature imply that the given test is not recent. **c** Features that represent the result of a laboratory variable and that are missing—are handled differently depending on the algorithm in question. For example, for XGBoost, which forms 46% (13 out of the 28 algorithms of CLL-TIM), missing values are fed directly without imputation as XGBoost automatically models missing data. For the remaining algorithms, median imputation is performed. In totality, imputation only ends up being employed for missing features of the routine laboratory variables, which are fed into non-XGBoost algorithms. **d** The final layer deals with a design that is robust to unseen missingness. Namely, ensembles are known to be robust to missingness and noise, particularly when the base-learners (i.e the algorithms making up the ensemble), have uncorrelated errors. CLL-TIM encouraged uncorrelated errors with the use of feature bagging, the use of different algorithms and parameters, and the use of different outcomes for different base-learners (see Methods—Algorithms, Feature and Variables in CLL-TIM). Lastly, CLL-TIM’s predictive confidence adds another layer of protection when predicting in new environments with missingness, as it can indicate when missingness is such that it is likely affecting predictive accuracy for a given patient. IGHV the immunoglobulin heavy chain gene, CRP C-reactive protein.

CLL-TIM was designed to handle missingness using several layers (see Methods and Fig. 6). Two aspects of missingness are notable between the research and deployment of EHR. In the research EHR, two out of the top three prognostic markers of CLL-TIM (Binet Staging and IGHV status) were available for 100% of patients but only available for 28% and 9% of the patients in the deployment EHR. Additionally, long-term laboratory data was generally not available in the deployment EHR (Fig.4 and Supplementary Fig. 2). We benchmarked CLL-TIM on these as-is missingness conditions that were not seen or anticipated in the development of CLL-TIM using Bench-B (*n* = 276) from the deployment EHR (Table 3). All missingness conditions were kept ‘as is’ and missingness was handled as described (Fig. 6). CLL-TIM on Bench-B achieved MCC = 0.500 (95% CI: 0.499–0.501) (high-confidence predictions) vs 0.56 (95% CI: 0.418–0.697) for CLL-TIM on the research EHR. Increasing the threshold for a high-confidence prediction from *t* = 0.58 to *t* = 0.65 (Bench C, *n* = 233 in Table 3) helped in improving the MCC further. However, such calibration should be subsequently tested for its performance on a new set of patients in the future.

**Fig. 7:**
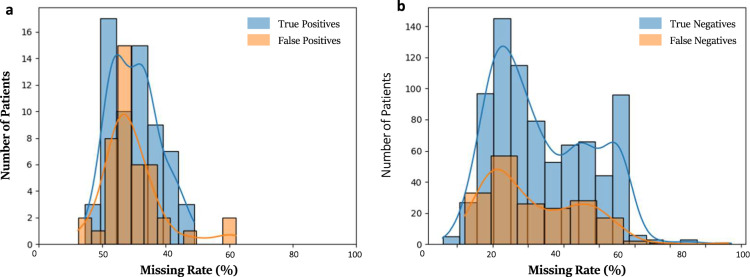
Performance of CLL-TIM with respect to missingness. Data are shown for CLL-TIM predictions on Bench-C (*n* = 1099). The missing rate for each patient is calculated as the percentage of missing features from CLL-TIM’s 228 features. **a** True Positives in blue and false positives in orange. **b** True negatives in blue and false negatives in orange. Missing rates show similar distributions for all patients irrespective of whether they were correctly or incorrectly predicted.

To assess how missingness in the deployment EHR may have affected CLL-TIM’s performance, we plotted the distribution of true-positives vs. false-positives with respect to the percentage of missing data for each patient, and similarly for true-negatives vs. false-negatives (Fig. 7). In both scenarios we found no marked differences in the missing rates for the correct or incorrect predictions. Our results suggest that CLL-TIM is robust to the general missingness of its variables but susceptible to missingness in its key variables, like the Binet Stage. The latter scenario is likely due to the fact that the Binet Stage was never missing during CLL-TIM’s development.

### Predictive confidence identifies trustworthy predictions

We next assessed whether on the deployment of EHR, the predictive confidence of CLL-TIM is a good indicator of which predictions are more likely to be correct over others (Supplementary Table 1). When not restricting to high-confidence predictions i.e., all predictions irrespective of confidence, CLL-TIM achieved a lower performance on Bench-A with MCC = 0.457 (95% CI: 0.453–0.460) vs. 0.715 (95% CI: 0.712–0.719) for high-confidence predictions. Similarly, on Bench-B, CLL-TIM achieved an MCC = 0.289 (95% CI: 0.288–0.290) when not restricted to high confidence predictions vs. 0.500 (95% CI: 0.499–0.501) for high confidence predictions only. Survival analysis on all predictions achieved a Hazard Ratio (HR) = 5.3 (95% CI: 2.46–11.42) vs. 11·06 (95% CI: 3.23–37.95) for high-confidence predictions only (Fig. 5). On Bench-B (*n* = 1099), 25% of CLL-TIM’s predictions were of high-confidence, and when controlling for the availability of key CLL-TIM prognostic markers, Binet Stage and IGHV mutation status (Bench A, *n* = 31), 83% of patients were predicted with high confidence.

### Prediction coverage of CLL-TIM and gold-standard in CLL

Besides generalization and trust, it is necessary for deployed models to be able to have a high prediction coverage. This is defined as the percentage of patients for which predictions can be made irrespective of missing data. CLL-TIM has high dimensionality (84 variables) but does not restrict the availability of all these variables for predictions to be available (see Methods and Fig.6). Hence, 100% coverage was achieved for CLL-TIM upon deployment. The gold standard in CLL prognostication, CLL-IPI^3^, had the following availability in its five variables (age: 100%, Binet Stage: 17.8%, β2 microglobulin: 64.8%, IGHV status: 18.9%, Del17p and/or TP53 mutation: <0.05%). Given that CLL-IPI requires that all variables are available for its calculation, only *n* = 7/1099 (0.63%) of patients in the deployment EHR could have a CLL-IPI score. When attempting to increase CLL-IPI’s prediction coverage by imputing two of its variables, Binet Stage and IGHV mutation status, the prediction coverage increased to *n* = 32/1099 (2.9%) of the deployment EHR.

## Discussion

**Fig. 8:**
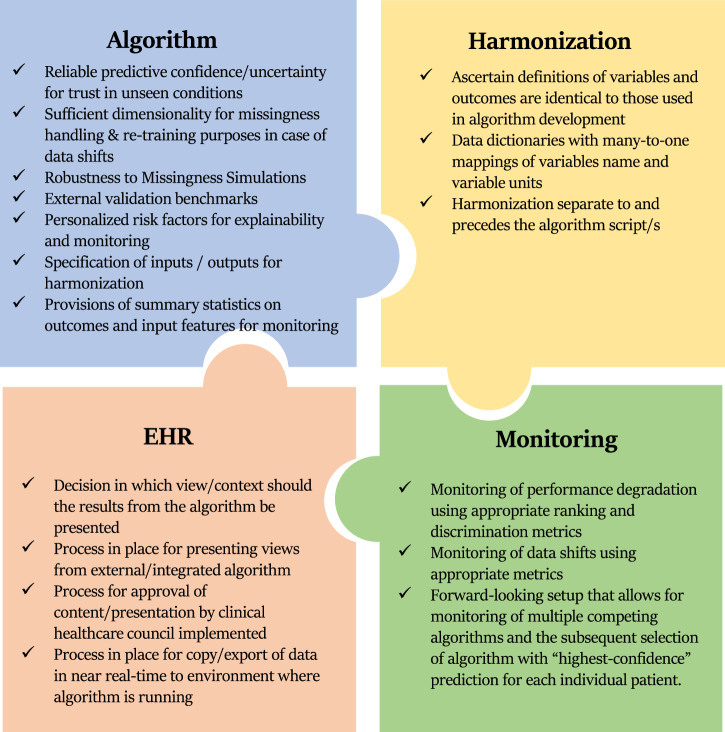
Checklist for deployment of algorithms into EHR. The following checklist summarizes the lessons learned from our real-world deployment of CLL-TIM into a production EHR.

Our work describes the practical challenges and processes for deployment of a high dimensional prognostic model adjoined to an EHR. While theoretical guidelines have been put forward^14,15^, proof-of-concepts of such implementations have been warranted. Our described algorithm deployment adjoined to an EPIC-based EHR, provides generalizable step-by-step guidance for the deployment of algorithms from research environments into EHR systems (Fig. 8).

For the deployment of a high-dimensional research algorithm like CLL-TIM, data harmonization was by far the most time-consuming aspect of this process. While the iterative method we devised may be replicated, automating such a process is not straightforward. Some variables required mapping by a physician researcher clinically experienced within the deployment EHR system, others required close interaction between data analysts familiar with the EHR system and those that designed CLL-TIM. The complexity of models that address issues of trust, robustness, fairness, privacy, and accuracy in prognostic models^4–9^ will likely require large-scale data harmonization across EHRs. With only 5% of prognostic models undergoing external validation^16^, further data harmonization efforts are needed.

Generalization of algorithms between cohorts is particularly difficult because of data shifts presenting situations to the algorithm that are unseen during its development^17^. In our case, several of CLL-TIM’s key variables, with no missingness in the research environment, had high missingness (72% missing clinical stage, 91% missing IGHV) in the deployment EHR. A workaround for the clinical stage and IGHV status to be manually entered into structured EHR as part of the staging process has been initiated. Additionally, the deployment of EHR had less historical data for laboratory and blood culture data. For the best generalization performance, besides using high-confidence predictions, it was sufficient to just match the condition of having an available clinical stage and IGHV status while allowing for the missingness of all other variables. General missingness had no marked effects on the correctness of predictions. However, predictions that include the patients with missing clinical stage and IGHV status reduced CLL-TIM ‘s performance through precision, but 95% confidence interval bounds of MCC and recall overlapped with those achieved by CLL-TIM on the research EHR, where these variables were 100% available. We attribute the adaptability of CLL-TIM to make predictions in high missingness scenarios, unseen during its development phase, to its ensemble design with high dimensionality i.e., its complexity. Diverse ensembles are known for their generalization properties^18^, and high dimensionality allows for risk to be calculated using different sets of variables for each new patient^10^. For example, because of missingness in the deployment EHR, the gold standard in CLL prognostication^3^, CLL-IPI, due to its low dimensionality (five variables), could only be calculated for 0.63% of patients. Even with high dimensionality, key variables, likely termed as prognostic markers, are Achilles heels as they increase the explainability/trust in a prognostic model from a clinician’s standpoint while reducing its ability to generalize to unseen missingness conditions. For example, in models that are consistently tested in changing conditions, neutralizing the effect of the most important variables was necessary for consistent generalization^19^. When thinking of deployment in several EHRs, the generalizability of an entire modeling pipeline, as opposed to a model, might also be preferred^20^.

Upon deploying a model on an unseen cohort of patients, predictions may fail or degrade over time^21^. Given that high-confidence predictions were generally more correct than low-confidence, in conditions of high-missingness, we could for each patient decide whether to trust their prediction depending on the assigned confidence. Improving the utility of our predictive confidence likely relies on understanding which factors help increase the percentage of patients for which a high-confidence prediction exists. In the development of CLL-TIM, we found that using long-term patient histories increased the percentage of high-confidence predictions. Here, we saw an increase in the percentage of high-confidence predictions when Binet Stage and IGHV mutation data were available. Thus, additional access to long-term data through the EHR and changing unstructured data (in this case, clinical stage and IGHV status) to structured data within the deployment EHR may improve performance without any modifications to the model itself. This is especially relevant since the FDA does not allow algorithm modification after approval^21^. Sustaining a high percentage of high-confidence predictions would likely require an ecosystem of competing prognostic models. For example, a low-confidence prediction by one model could be overridden by another competing prognostic model with a high-confidence prediction for that same patient. Despite its utility in health applications, predictive confidence remains mostly unused in prognostic models^2,22^.

Our framework for monitoring is designed to detect drift in model performance but also any data shifts in the outcomes, inputs, and personalized risk factors. This enables us to anticipate and address a potential performance drift in the model before the respective 2-year outcomes may be extracted. Additionally, our setup allows us to ascertain that predictions are correct for explainable reasons. The next steps in our monitoring set-up will focus on the types of interventions we can perform to regain model performance if it degrades, such as re-training. Note that if degradation is due to missingness in, say, two variables from a five-variable prognostic model, re-training a now three-variable model is still unlikely to improve performance. Therefore, for sustained predictive performance upon deployment, one would either need sufficient dimensionality for successful re-training or a framework that can switch to alternative prognostic models when a low-confidence prediction occurs.

We propose that for algorithms that seek external validation and deployment into new EHR systems that may present unseen missingness conditions, high-dimensional algorithms with handling of missingness and reliable confidence are preferred over low-dimensional algorithms without. Good handling of missingness should include robustness measures during the development and training phases of an algorithm. For example, data augmentation by down-sampling portions of a patient’s EHR by purposely removing parts of it^23^. Additionally, results on any internal and external validation should be accompanied by a performance assessment under several missing data simulations. For example, in CLL-TIM’s development, we assessed results when no long-term histories were present and when all the variables of a given data modality (e.g., baseline variables, laboratory blood cultures, pathology, and diagnosis) were purposely removed in the prediction phase^10^.

While during the last 5–10 years, there has been a substantial increase in the number of algorithms approved within medical device regulations in the US and Europe, the legal process is not clear. Parts of the approval system are decentralized and non-transparent^24^. Despite guidelines and recommendations on the implementation^15^ of algorithms into EHR, most approved algorithms do not follow these^25^. This may reflect a disconnect between administrators, health authorities, clinical researchers, and data scientists who develop the algorithms. This, whether commercially or academically based. Thus, we here demonstrated the necessary framework of stakeholders and their roles and a path for academic deployment of algorithms that does not require a resource-demanding CE labeling through the Medical Device Regulation path^26^.

In conclusion, design choices during prognostic model development can significantly impact their suitability for clinical use, especially when deploying them to different EHRs. High-dimensional models, designed to also handle missingness, are better able to sustain predictive accuracy in situations with missing data and are more amenable to handle future data shifts if retraining is needed. Reliable confidence estimates accompanying predictions are crucial for trust when integrating research algorithms into new EHR environments. Overcoming the bottlenecks of data harmonization across EHRs is a key hurdle in deploying more data-driven algorithms in clinical settings. The successful deployment of the high-dimensional CLL-TIM model offers a proof-of-concept for open-source, non-commercial algorithm adoption in EHR systems, providing a roadmap for others looking to do the same.

## Methods

### CLL-TIM summary

CLL-TIM was developed as an ensemble model integrating 28 different machine-learning algorithms, trained on data from 4149 patients with CLL as previously described^10^. CLL-TIM identifies patients with a high risk (precision of 72% and a recall of 75%) of serious infection and/or CLL treatment within two years from the time of diagnosis (Fig. 1a). The research database used for the development of CLL-TIM referred here as the research EHR, was assembled based on a combination of data from the CLL registry and routine laboratory data from the biochemistry, microbiology and pathology systems. These were collected and structured through the PERSIMUNE data warehouse (persimune.org)^27^ as previously described^10^.

### Algorithms, features, and variables in CLL-TIM

CLL-TIM’s 28 algorithms (called base-learners) include 13 XGBoost, seven Random Forest, 4 Extra Trees, 2 Elastic Network, and 2 Logistic Regression models. Twenty of these, model the composite outcome, five are specific to the CLL treatment outcome and three are specific to the infection outcome. While 7288 features spanning 620 variables were used during the development of CLL-TIM, the feature selection, and automated selection of CLL-TIM’s base-learners (including feature bagging), resulted in a final data-driven set of 228 features that spanned 84 variables. For example, a single variable, like platelets, may have one feature that encodes the minimum value over the last 3 months and another feature that encodes the median value over the last 7 years.

### Calculation of CLL-TIM confidence

Confidence of CLL-TIM is a numerical value ranging from 0 to 1, where 0 indicates CLL-TIM is most confident of a given low-risk prediction, and 1 indicates it is most confident of a given high-risk prediction. Values close to 0.5 indicate that CLL-TIM is unsure. This confidence is derived using soft-voting over all of CLL-TIM’s 28 algorithms, where the probabilistic output of each algorithm is averaged to get the CLL-TIM confidence. The rationale here is that the confidence represents agreement or disagreement between the several algorithms of CLL-TIM. During the development of CLL-TIM, cutoffs for what is considered high-risk with high-confidence were set at ≥0.58, and ≦0.28 for low-risk high-confidence predictions. Anything between 0.58 and 0.28 was considered a low-confidence prediction. The cutoffs were chosen based on the PreVent-ACaLL criteria^13^, which required that 20% of the predictions be high-risk with high-confidence and 30% of the predictions be low-risk with high confidence^10^. On the research EHR, on a separate cohort from which the cutoffs were generated, CLL-TIM’s high-confidence predictions had a hazard ratio (HR) of 7.27 compared to a HR = 2.09 for low-confidence predictions. This confirmed the use of averaging the probabilistic output of CLL-TIM’s base-learners, as a reliable indicator for which predictions are more likely to be correct over others.

### Handling of missingness in CLL-TIM

Due to its various missingness handling mechanisms in place, while CLL-TIM is trained using 84 variables and 7-year patient histories, it does not require them to be available for all patients. CLL-TIM addresses missingness over three layers (feature, algorithm, ensemble) that are designed to both handle missingness and to be robust to future missingness (Fig. 6). Missingness at the feature layer is addressed by encoding certain features, such as routine laboratory variables and baseline variables, to indicate when data is missing. This allows algorithms to adjust their decisions based on feature availability. On the algorithm layer, missing values for certain features vary depending on the algorithm. XGBoost (13/28 algorithms of CLL-TIM), for example, handles missing data ‘as-is’ internally^28^, while other algorithms necessitated median imputation. On the ensemble layer, we performed ensembling with feature bagging that is conducive to better generalization performance in situations of unseen noise and missing data^18,29^—for example, those when using CLL-TIM in new EHRs, hospitals and countries. Additionally, in such unseen scenarios, CLL-TIM’s confidence can be used as a reliable indicator of whether to trust a given prediction.

### Data harmonization and matching predictions across deployment EHR

Data harmonization is essential for integrating an AI algorithm into a new EHR cohort. The central task involves aligning variable nomenclature and units with the algorithm’s specifications. For this, we created two many-to-one mapping dictionaries. One for naming conventions and the other for unit conventions (Fig. 2). A physician researcher specialized in hematology selected and matched variable labels. To ascertain the validity of our mapping dictionaries, we devised an iterative process that, for an identical group of patients, compares CLL-TIM’s predictions, when their data comes from the research EHR, and when it comes from the deployment EHR. The mapping is considered finished when for a given patient, CLL-TIM’s predictions match on both EHRs. Further details of the described data harmonization may be found in the Supplementary Methods.

### Automation of CLL-TIM predictions into the deployment EHR and monitoring

We set up CLL-TIM as a standalone script that accepts patient data and produces for each patient a risk level, confidence in the prediction, and a set of personalized risk factors (top five factors pushing towards high-risk and top five pushing towards low-risk). The script is ‘plug-n-play’, in that it can be run on any system as long as python is available and raw patient data (not features) are entered in the right format. Automation into EHR, therefore, focused mostly on how to automate the process of importing data into and exporting results from CLL-TIM’s standalone script (Fig. 3). Patient data fed into CLL-TIM is stored in the Chronicles database, with nightly ETL processes transferring data to Clarity and Caboodle databases. The previously described harmonization scripts pre-process data for CLL-TIM, then CLL-TIM is executed to provide predictions and its results are then integrated back into the Chronicles EHR database. The latter enables the provision of CLL-TIM’s results within the EHR interface (Fig. 3). This design, featuring containerization and shared databases for predictions and patient data, supports centralized monitoring and streamlined access of both CLL-TIM’s outputs (to assess performance degradation) and CLL-TIM’s inputs (to assess data-shifts)—Supplementary Fig. 3. In our setup, the hospital has full control and rights over the environment where CLL-TIM is executed, without any licensing or permissions required from the commercial provider of the deployment EHR. In light of potential model degradation, close monitoring of prognostic model performance after clinical deployment is crucial^21^. Our monitoring setup for CLL-TIM, upon its deployment, involves automatic tracking of outputs and outcomes for each patient through the caboodle database. Every three months, we evaluate using several metrics, both predictive performance decay and data shifts, that may be responsible for the deterioration of predictive power (Supplementary Fig. 3 and Supplementary Methods).

### Stakeholder roles and legal framework

For delivering CLL-TIM’s results to treating physicians within the deployment EHR system, a multidisciplinary team including researchers, analysts, IT experts, interface developers, clinical council, and business owners collaborated (Table 1). This ensured comprehensive consideration of clinical, technical, and regulatory aspects. To ensure that the deployment of CLL-TIM met the requirements for a decision support tool, a Medical Device Regulation (MDR) assessment was performed according to Medical Device Regulation (EU) 2017/745 paragraph 5.5^26^. The algorithm was considered a class 2 A device according to annex 8, rule 11 (software which is intended to provide information that is used to make clinical, diagnostic, or therapeutic decisions, except if leading to decisions that can cause serious harm or surgical interventions, the latter resulting in classification 2B). As CLL-TIM was only implemented into one EHR system within a limited organization, the system could be implemented locally without a resource-demanding CE labeling through the Medical Device Regulation path. Self-evaluation according to the guidelines on MDR evaluation from the local institution was put in place to meet the requirements of paragraph 5.5 for the local implementation of a 2A medical device. Before presenting CLL-TIM’s predictions to practicing physicians on the EHR interface, an assessment was performed by the Hematological Clinical Healthcare Council. The latter is responsible for the assessment of any significant changes to the EHR interface that may impact the management of patients with hematological disorders. The council requested proof of (i) the mapping of all relevant mapping within the caboodle copy of the EHR, (ii) confirmation and validation of all parts of the script and predictions within the EHR, (iii) validation of all predictions for patients matched between the research and deployment EHRs, (iv) validation of the personalized high/low-risk factors for these matched patients, and (v) training of all relevant physicians on interpretation and use of CLL-TIM as a decision support tool. As CLL-TIM is still being tested in a clinical trial, the predictions were furthermore labeled as “research-use only” within the deployment EHR.

### Data approval

Based on approval from the National Ethics Committee and the Data Protection Agency, EHR data and data from health registries for current and previous patients with CLL were retrieved as previously described^10,30^.

### Supplementary information

Supplementary Material

## Data Availability

The individual patient-level data that support the findings of this study are available from the corresponding author upon reasonable request. As the individual patient-level data cannot be anonymized, only pseudonymized, according to Danish and EU legislation, the data cannot be deposited in a public repository. However, the authors provide a data repository with individual patient level data that can be made available with 2-factor authentication for researchers on a collaborative basis upon request.

## Code availability

CLL-TIM code is available in the supplementary.
