# Supplementary material for: Deployment and validation of the CLL treatment infection model adjoined to an EHR system
Source: NPJ Digit Med. 2024 Jun 5;7:147. doi: 10.1038/s41746-024-01132-6 (PMC11153589; doi:10.1038/s41746-024-01132-6)
Supplement: Supplementary file 1 — Supplementary Material [file 41746_2024_1132_MOESM1_ESM.pdf]

Supplementary Material

Deployment and validation of the CLL treatment infection model adjoined to an EHR system - Proof of concept deployment of data-driven decision support

Supplementary Methods

Data Harmonization & Matching Predictions across production EHR

Data harmonization is necessary for implementing an AI algorithm into a new EHR cohort. This usually requires the process of feeding the right variables in a format, that complies with the conventions and units that are defined by the AI algorithm. The challenge is that a single variable may have different naming conventions in the EHR. For example, in the EHR cohort described in this work, uric acid is represented as both "URAT (MMOL/L);P" and "URAT;P". Effectively the task of data harmonization is that of creating a dictionary of many-to-one mappings, that translate several EHR variable instances to a single variable instance, as defined by the algorithm in question (Fig. 2). For implementation of CLL-TIM from a research EHR into the EHR production database (deployment EHR) we therefore required i) mapping of the input variables across databases and ii) matching of CLL-TIM's predictions for an identical group of patients from both databases. The latter step is not always necessary, as it is predicated on having a set of patients that are common to both the research and EHR cohorts, and may therefore be skipped if not available. For mapping CLL-TIM variables within the EHR, initially a list of variables with labels matching the labels for the 84 original variables included for the final version of CLL-TIM was produced. A physician researcher specialized in hematology that had clinical experience treating patients with CLL within this EHR system, assessed the list of variables from the EHR and selected and matched the appropriate variable labels. For a few variables (IgHV mutational status, clinical Binet stage, ECOG performance status and cytogenetic aberrations tested by FISH analysis), structured data were not directly accessible through the EHR; these variables had been supplied to the research database by collection from the CLL register, while results for these variables were provided as pdf files within the EHR. To provide these variables as structured information for the harmonization process of CLL-TIM, the staging system of the EHR was updated to include these variables in structured form. It was requested by the treating physicians to enter these data at the first follow up visit after diagnosis for all patients with a diagnosis of CLL. These now structured data will subsequently be used to automatically feed into the mandatory reporting for the national CLL register.[2] For matching the input variables between the research and the deployment EHR, we created two dictionaries. One dictionary was used for the mapping of variable names from the deployment EHR database to those used in the research EHR. Some of the variables required many-to-one mappings, as in the deployment EHR, different nomenclatures could be referring to the same CLL-TIM variable. A second dictionary was used for the mapping of units for routine laboratory tests. This included also many-to-one mappings due to the different ways the same units were presented in the deployment EHR. Successful remapping of variables' names and units means that thereafter, for running CLL-TIM in the deployment EHR, we could use the original CLL-TIM code without any further changes

After manual mapping of CLL-TIM variables, we next started the iterative process that in its finality, arrives at matched predictions, for the same set of patients for both the research and deployment EHRs (Fig. 2). Since variables from the deployment EHR have a many-to-one mapping to those in the research EHR, not all variables in the previous manual mapping phase may have been identified. Additionally, within the deployment EHR, the naming conventions of variables and their respective units of measurements were not necessarily identical to those in the research EHR. Effectively we employed an iterative process of running predictions of CLL-TIM on the research EHR in parallel to the deployment EHR, on an identical group of patients. For each patient with discrepancies in the predictions across EHRs, troubleshooting was performed to assess the mapping of variables from the deployment EHR. As an additional check, besides comparing the numerical value of the predictions across EHRs for a given patient, we also checked that their personalized risk factors matched. This ensured that both CLL-TIM, on both EHRs, was for the same patients, arriving at the same prediction using the same features and their contributions.

Setup for CLL-TIM Performance Monitoring on the Deployment HER

It is well-known that model performance can degrade over time[1]. This can be due to changes in the data population encountered by the predictive algorithm, including but not limited to changes in, i) medications, ii) general treatment approaches, iii) demographics of the patient population, iv) the facility structure and/or referral

processes, v) the disease landscape (consider the COVID pandemic as an example), vi) practice of laboratory tests performed, vii) laboratory procedures resulting in different routine results. It is therefore of vital importance to closely monitor prognostic model performance after the model has been put into clinical use. Our setup for CLL-TIM monitoring is depicted in Supplementary Fig. 3. Firstly, our setup is such that monitoring may be done automatically, since all outputs from CLL-TIM (i.e risk level, confidence, personalized risk factors) calculated for each patient are saved within the caboodle database, together with the input data. All future outcomes (initiation of CLL treatment or infection defined as a blood culture drawn) for the patients, are linked to CLL-TIM outputs for the respective patients through the Danish unique identification number. Our proposed setup, monitors both the degradation of predictive performance (Performance Monitoring, Supplementary Fig. 3) and data-shifts that may be the cause of the degradation (Data-shift Monitoring, Supplementary Fig. 3). The aim is to have the monitoring executed once every 3 months. For performance monitoring, we check CLL-TIM's ability to rank individuals with events, higher than those with no events, using the Precision-Recall Area Under Curve (PR-AUC). Given that the aim of CLL-TIM is however to be used in a classification scenario, and not for relative risk ranking, we also monitor its ability to classify patients using the Matthew's Correlation Coefficient (MCC), together with the precision, recall and other metrics. For data-shift monitoring, firstly we aim to monitor the outcomes using cumulative plots to check whether the relative frequency of outcome events are changing over time. For example, changes in recall of CLL-TIM may be due to lower incidence of infections in the general population. Univariate testing on CLL- TIM's features enable us to detect data-shifts caused by a multitude of sources including human input error. Though not the case for this EHR deployment, this is particularly important in scenarios where patient data are manually entered, as we observed in the ongoing clinical trial using CLL-TIM[2]. For the EHR deployment, the monitoring of input data-shifts will mainly focus on laboratory tests and changes in the results provided by the laboratories, say upon changes in analysis equipment. In CLL-TIM's development[3] we observed that high-risk individuals may have completely different risk factors, and therefore there are many "pathways" that may lead to a high-risk individual. We observed this heterogeneity through clustering of the top risk factors that co-occurred together. We aim to use this same clustering at each monitoring iteration and assess any changes in the most populated risk factor clusters. For example, if Binet stage commonly shows up in a key number of clusters, but stops showing up in subsequent iterations, we may use this information to assess whether a change has happened with how Binet Stage is being fed into CLL-TIM. This, in turn enables us to target such a potentially problematic variable. Through the provision and monitoring of personalized risk factors, we are thus able to link the potential degradation in performance to specific errors or changes in the input data.

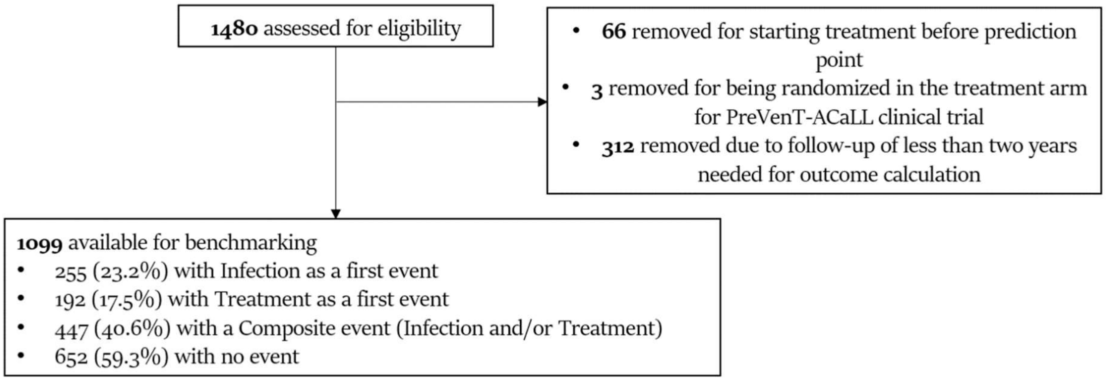

Supplementary Figure 1. Consort Diagram. 1480 patients diagnosed with CLL in Denmark post 2018 were eligible for retrospective benchmarking of CLL-TIM in this work. CLL-TIM was developed using patients diagnosed prior to 2018 and therefore this benchmark presents a non-overlapping set of patients. Prediction point refers to three-months post-diagnosis. All available patient data up until prediction point is used for predicting the two-year risk composite outcome of infection and/or treatment post prediction point. The allocation of 'no event', 'Infection as a first Event', 'Treatment as a first Event' and 'Death as a first Event', were all restricted to events within the two-year predictive window.

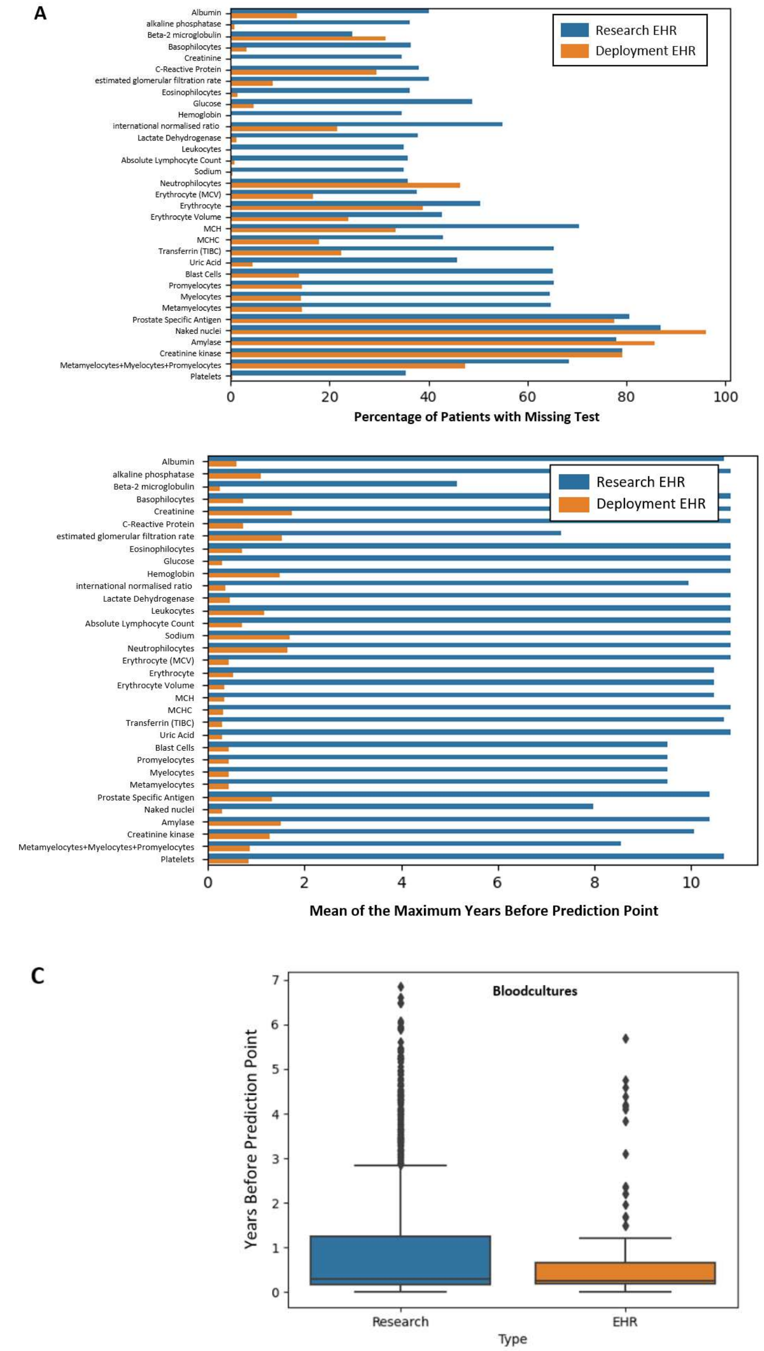

Supplementary Figure 2. Comparison of Missingness for Laboratory and Blood Cultures between Research and EHR cohorts. A Percentage missing rate of laboratory data. Missing rate is calculated as the rate of patients with no laboratory test in the respective cohort. For 57% of the laboratory tests, the rate of missingness is higher for the EHR cohort to the Research cohort. **B Historical look-back for laboratory data.** For each laboratory test we calculated for each patient, the earliest test available before the time of CLL diagnosis. For each laboratory test, we then took the mean over all patients of the earliest test available. This gives an indication on how far-back each laboratory test is available. From this we could observe that the research EHR has longer historical data than the deployment EHR cohort **C. Historical look-back for blood cultures.** Shown is the distribution of when blood cultures were taken for both cohorts. Boxplot shows the three quartile values of the distribution and extreme values. Whiskers extend to the 1.5 IQRs of the lower and upper quartile, and extreme values are those which fall outside this range. The extremes are displayed independently for each data point.

16.56% and 6.7% of patients had at least one blood culture history available for the research and EHR cohorts respectively. TIBC – Total iron-binding capacity, MCV – Mean cell volume, ECOG - Eastern Cooperative Oncology Group. MCHC - mean corpuscular hemoglobin concentration, MCH - mean corpuscular hemoglobin.

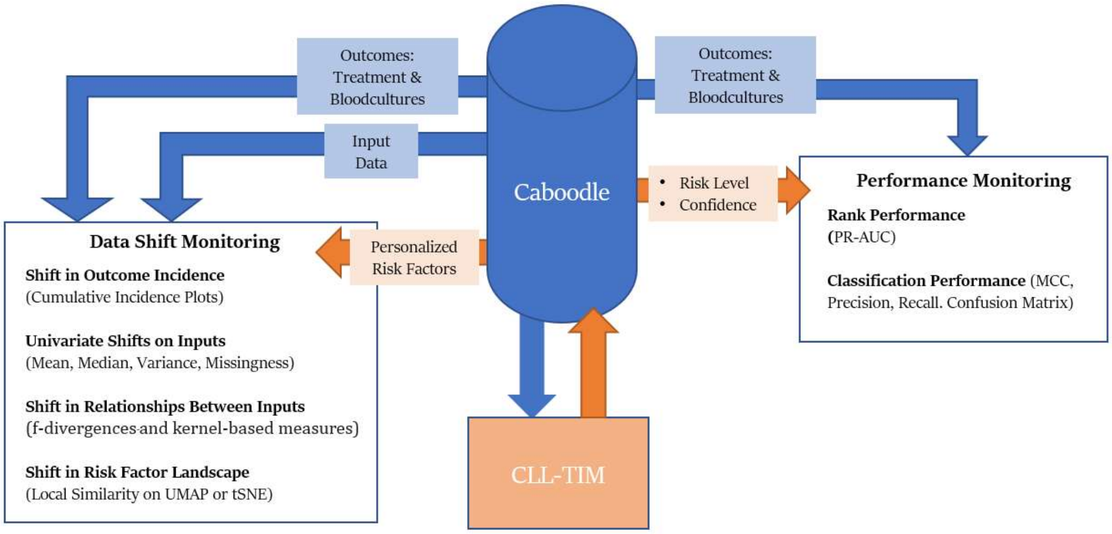

Supplementary Figure 3. Setup for CLL-TIM Monitoring. Our setup for algorithm monitoring is aimed at detecting drift in performance but also at understanding the potential reasons why a potential drift is seen. For these reasons, we assess changes in both performance of CLL-TIM, i.e. how well it's predictions represent the ground truth (Performance monitoring), and also changes in the data that CLL-TIM uses for its predictions (Data-shift monitoring). Patient Data, outcomes and CLL-TIM outputs are all stored on the caboodle database making it easy to run both performance and data-shift monitoring within the same script. For data-shift monitoring we use cumulative incidence plots to check for any changes in the rate of infections and CLL treatments. For all 228 features of CLL-TIM, we calculate univariate statistics related to their values and missingness. As an additional check, we also aim to monitor the relationships between inputs, for this we use methods (f-divergences and kernel-based measures) aimed at high dimensionality[15]. Finally, we can also assess whether the "reasons for prediction" are also changing by looking at changes in the risk factor landscape. Namely given CLL-TIM uses different risk factors to predict the risk of individuals, in CLL-TIM we performed clustering analysis using UMAP and tSNE of the different combinations of co-occurring risk factors that were used to predict individual risk. Given such clusters for CLL-TIM, we can measure the similarity of clustering at each monitoring iteration to check for any drift in the risk factors responsible for predicting the risk of the given population. For performance monitoring, we assess the ranking performance of CLL-TIM using the PR-AUC and the discrimination performance using several other metrics. For our outcome of interest (treatment and/or infection), we see an incidence rate of less than 50% which is why we opt for the PR-AUC and MCC over AUC and Accuracy. The latter two metrics give biased results when outcome data is imbalanced. MCC – Matthew's Correlation Coefficient, PR-AUC – Precision-Recall Area Under Curve. UMAP - Uniform Manifold Approximation and Projection. tSNE - t-distributed stochastic neighbor embedding.

		Research EHR (n=288)	Deployment EHR BENCH-A (n=47)	Deployment EHR BENCH-B (n=1099)	Deployment EHR BENCH-C (n=1099)
Missingness Control		CLL-IPI variables non-missing	Binet Stage and IgHV non-missing (two of the CLL-IPI variables)	As-is missingness	As-is missingness
% Availability	Binet Stage<br>IgHV<br>β2M<br>ECOG Status<br>FISH status<br>Other CLL-TIM Variables	100%<br>100%<br>100%<br>100%<br>100%<br>Varied	100%<br>100%<br>64%<br>0%<br>0.05%<br>Varied	28%<br>9%<br>64%<br>0%<br>0.05%<br>Varied	28%<br>9%<br>64%<br>0%<br>0.05%<br>Varied
Metrics	PR-AUC	0.662 (0.582-0.741)	0.928 (0.927-0.929)*	0.443 (0.442-0.444)*	0.443 (0.442-0.444) *
	MCC	0.414 (0.3-0.525)	0.457 (0.453-0.460)	0.289 (0.288-0.290)*	0.286 (0.285-0.287) *
	Precision	0.629 (0.545-0.718)	0.856 (0.854-0.858)*	0.484 (0.484-0.485)*	0.597 (0.595-0.598)
	Recall	0.568 (0.462-0.663)	0.642 (0.640-0.645)	0.392 (0.391-0.392)*	0.252 (0.251-0.253)*
	TP	54	18.621 (18.548-18.693)	98.675 (98.459-98.890)	63.493 (63.303-63.683)
	FP	32	3.192 (3.148-3.236)	105.159 (104.890-105.428)	43.018 (42.842-43.194)
	TN	161	14.808 (14.764-14.852)	741.841 (741.572-742.110)	803.982(803.806-804.158)
	FN	41	10.379(10.307-10.452)	153.325 (153.110-153.541)	188.507 (188.317-188.697)

Supplementary Table 1. **Comparative Benchmarking of Low-Confidence Predictions of CLL-TIM on Deployment EHR vs Research EHR** Predictions for Research EHR were assessed on an internal research benchmark during the development of CLL-TIM. Namely, a subset of patients in test cohort with full CLL-IPI and a full two-year follow-up. All patients in this set were diagnosed with CLL prior to 2018. For deployment EHR, patients were diagnosed with CLL post 2018 and therefore were not part of the development of CLL-TIM. Prediction point was at three months post CLL diagnosis and the binary outcome predicted was whether a patient had an infection or needed CLL treatment within 2 years from the prediction point. For this classification analysis, patients with no event and a follow-up of less than 2 years were removed from the analysis (for analysis with these patients included through censoring, please see Kaplan-Meier curves in Fig. 5b). All predictions shown are for all patients irrespective of the confidence level that CLL-TIM predicted the risk with – this includes both high-confidence and low-confidence predictions. Results for the Research EHR are taken from original publication of CLL-TIM and calculated on patients for which CLL-IPI variables were completely available. Bench-A - For an equitable comparison we present results for CLL-TIM on the deployment EHR, on the subset of patients with available Binet Stage and IgHV status. Bench-B – shows performance on all patients with any missing data. Bench-C - Identical benchmark to Bench-B, except that we tested the use of calibration. Namely we increased the threshold of what is considered to be high-risk from 0·5 (derived from original CLL-TIM publication) to 0·6. PR-AUC - Area under Precision-Recall Curve, MCC - matthew's correlation coefficient, TP, FP, TN, FN are true-positive, false-positive, true-negative, false-negative predictions for two-year outcome. IgHV - the immunoglobulin heavy chain gene, β2M - β2 microglobulin, FISH - DNA fluorescence in situ hybridization, ECOG - Eastern cooperative oncology group. Results are for 5,000 bootstrapped patient cohorts where sampling was performed with replacement and stratified to preserve original high-risk to low-risk patient ratios within each bootstrapped dataset. We then generated 95% confidence intervals (shown in brackets) for each metric over the bootstrapped datasets. * Indicates that difference between the measurements (Research EHR vs Bench A, B or C) is statistically significant as 95% confidence intervals do not overlap. Comparison was limited to PR-AUC, MCC, Precision and Recall.

CLL-TIM standalone python script

```
# -*- coding: utf-8 -*-
"""
Created on Tue Jul 25 14:24:43 2023

@author: Ragius
"""

import os

import pickle
import pyodbc
import keras
import pandas as pd
import numpy as np
import tensorflow as tf
from sklearn.preprocessing import MinMaxScaler
from revoscalepy import RxInSqlServer, RxSqlServerData, rx_import
############### Load lab results and cancer staging from reporting tables in Caboodle
# Deserialize the sys.tables result
labdata = pickle.loads(ExtraInput1)
# Initialize input data to numpy format
data_in = np.array(my_input_data)
# Load pickle file
cwd = os.getcwd()
os.chdir("C:\Custompackages\AIX")
cwd_temp = os.getcwd()
os.chdir(cwd)
#################


"""
import xgboost as xgb
import pickle
import shap
import numpy as np
import pandas as pd
import scipy
from sklearn import preprocessing
from datetime import time,timedelta
from datetime import date
import warnings
warnings.filterwarnings(module="sklearn*", action="ignore", category=DeprecationWarning)
warnings.filterwarnings(module="sklearn*", action="ignore", category=RuntimeWarning)
warnings.filterwarnings(module="sklearn*", action="ignore", category=UserWarning)
warnings.filterwarnings(action="ignore")
# SCALING AND NAN TO MEAN FUNCTIONS
def Nan2Mean(b):
    a = np.copy(b)
    # Nan to mean and Scaling
    col_mean = np.nanmean(a, axis=0)
    col_mean[np.isnan(col_mean)] = 0
    if np.sum(~np.isfinite(col_mean)) > 0:
        col_cnt = 0
        for c in col_mean:
            if np.logical_or(str(c) == "-inf", str(c) == "inf"):
                miny = np.nanmin(a[np.isfinite(a[:, col_cnt]), col_cnt])
                maxy = np.nanmax(a[np.isfinite(a[:, col_cnt]), col_cnt])
                Listy = np.argwhere(np.logical_and(~np.isfinite(a[:, col_cnt]), ~np.isnan(a[:, col_cnt])))
                for l in range(np.shape(Listy)[0]):
                    if str(a[Listy[l][0], col_cnt]) == "-inf":
                        a[Listy[l][0], col_cnt] = miny
                    elif str(a[Listy[l][0], col_cnt]) == "inf":
                        a[Listy[l][0], col_cnt] = maxy
            col_cnt = col_cnt + 1
    col_mean = np.nanmean(a, axis=0)
    col_mean[np.isnan(col_mean)] = 0
    # Find indicies that you need to replace
    inds = np.where(np.isnan(a))
    a[inds] = np.take(col_mean, inds[1])
    return a, col_mean
def Nan2Mean_useColmean(b, col_mean):
```

```
    a = np.copy(b)
    inds = np.where(np.isnan(a))
    a[inds] = np.take(col_mean, inds[1])
    return a

today = date.today()
todayF1 = today.strftime("%Y-%m-%d")  # used as prediction point
############################
# READING DATA
############################
DATA_LOCATION=r"C:/CustomPackages/AIX/"
PAll_Data = labdata
PAll_Basic = my_input_data


####################
# CONSTANTS
####################

# Units Format Conversion
Units = {"µmol/L": "mcmol_L",
       "µmol/l":"mcmol_L",
        "mmol/L": "mmol_L",
        "mmol/l":"mmol_L",
        "x 10E9/L": "10_9_L",
        "10E9/L":"10_9_L",
        "10E9/l":"10_9_L",
        "× 10":"10_9_L",
        "× E9":"10_9_L",
        "E9/L": "10_9_L",
        "× 10<sup>9</sup>":"10_9_L",
        "g/L": "g_L",
        "g/l": "g_L",
        "mg/l":"mg_L",
        "mg/L":"mg_L",
        "µg/L": "mcg_L",
        "E12/L": "10_12_L",
        "10E12/L":"10_12_L",
        "× E12</sup":"10_12_L",
        "x 10E12/L":"10_12_L",
        "mL/min": "mL_min",
        "ml/min": "mL_min",
        "mL/min1.73m2": "mL_min",
        "U/L": "U_L",
        "U/l": "U_L",
        "fL": "f_L",
        "fl": "f_L",
        "fmol": "fmol"
        }


# Mapping
LabelConv = {"IGHV mutation": "ighv",
             "IGHV": "ighv",
             "del13q14 mutation": "13q",
             "del11q mutation": "11q",
             "CD38 Immunophenotyping": "cd38",
             "del17p mutation": "17p",
             "Trisomy 12 mutation": "tri12",
                    "Binet staging system": "binet",
                    "KREATININ (ENZ.);P": "crea",
                    "LEUKOCYTTER;B": "wbc",
                    "TROMBOCYTTER;B": "plat",
                    "ERYTROCYTTER;B": "rbc",
                    'ERYTROCYTTER: VOL.FR.;B': "hema",
                    "TRANSFERRIN;P": "trans",
                    'PSA TOTAL;P': "psa",
                    'METAMYELO.+MYELO.+PROMYELOCYTTER;B':'mic',
                    'LYMFOCYTTER;B': 'pblympho',
                    'LYMFOCYTTER (MIKR.);B': 'pblympho',
                    'AMYLASE, PANCREASTYPE KAT.K.(IFCC 2006);P': 'amy',
                    'BASOFILOCYTTER;B': 'baso',
                    'BASOFILOCYTTER (MIKR.);B': "baso",
                    'EOSINOFILOCYTTER;B': 'eos',
                    'EOSINOFILOCYTTER (MIKR.);B':  'eos',
```

```
                'NEUTROFILOCYTTER (MIKR.);B': 'neutro',
                'NEUTROFILOCYTTER;B': 'neutro',
                'KREATINKINASE;P': 'creak',
                'GLUKOSE;P': "gluc",
                'NØGNE KERNER;B': "NØGNE KERNER;B",
                'METAMYELOCYTTER;B': "metamyel",
                'BLASTCELLER(USPEC.);B': "blast",
                'ALBUMIN;P': "alb",
                'HÆMOGLOBIN [MCHC];ERC(B)': "mchc",
                'BASISK FOSFATASE;P': "alp",
                'SMUDGE CELLER;B DNK35269': "smudge",
                'ERYTROCYTVOL. MIDDEL [MCV];ERC(B)': "mcv",
                'MYELOCYTTER;B': "myel",
                'TRANSFERRIN;P': "trans",
                'EGFR/1:73M²(CKD-EPI);NYRE': "egfr",
                'EGFR / 1:73M²(CKID: CYSC)(BØRN <18 ÅR)':"egfr",
                'EGFR/1,73M²(CKD-EPI);NYRE':"egfr",
                'BETA-2-MIKROGLOBULIN (MG/L);P': "b2m",
                'KREATININ (ENZ.);P': "crea",
                'LAKTATDEHYDROGENASE;P': "ldh",
                'PROMYELOCYTTER;B': "prom",
                'URAT (MMOL/L);P': "urat",
                'URAT;P': "urat",
                'C-REAKTIVT PROTEIN [CRP];P': "crp",
                'HÆMOGLOBININDHOLD [MCH];ERC(B)': "mch",
                'NATRIUM;P': "na",
                'KOAGULATIONSFAKTOR II+VII+X [INR];P': "inr",
                'HÆMOGLOBIN;B': "hbm",
    }

# Variable Name Translation Lists
RCLabList = ["na", "alb", "alp", "inr", "b2m", "wbc", "plat", "rbc", "hbm", "urat", "baso", "trans", "psa", "smudge", "amy", "pblympho", "mic", "mcv", "prom", "crp", "gluc", "crea", "creak", "hema",
        "egfr", "eos", "ldh", "neutro", "mch", "mchc", "blast", "myel", "metamyel"]
LabList_Orig = ["NA+", "ALB", "ALP", "INR", "B2M", "LEUK", "THR", "NPU01960", "HAEM", "NPU03688", "BAS", "NPU03607", "NPU08669", "NPU17597", "NPU19653", "LYM", "NPU26631", "NPU01944", "NPU03974", "CRP", "GLUC", "CRE", "NPU19656", "NPU01961",
        "EGFR", "EOS", "LDH", "NEU", "NPU02320", "NPU02321", "NPU03972", "NPU03976", "NPU03978"]
RCBaselineList = ["binet", "ighv", "ecog", "famcll", "agereg", "Beta2m", "cd38", "zap70", "sex", "13q", "tri12", "11q", "17p"]
BaselineList_Orig = ["binet", "umut", "WHOPERFORMANCE", "FAMCLL", "Age", "beta2m", "CD38", "ZAP70", "Gender", "del13", "tri12", "del11", "del17"]
################################################################################
# Data Loading:Loading Feature Decoding Information and Training Data for CLL-TIM
################################################################################
CurrEnsSize = 28
indexXGB = [0, 2, 3, 5, 7, 10, 11, 12, 15, 19, 21, 24, 25]
TransList = np.load(DATA_LOCATION+"TrainsListing.npy")
LabbiesNames = pd.read_csv(DATA_LOCATION+"LabTestFeatStruc.csv", sep=",", float_precision="round_trip")
TrainMatrix = np.load(DATA_LOCATION+"DTM.npy")
Collect_CVData_Chosen =np.load(DATA_LOCATION+"Collect_CVData_Chosen.npy")
Collect_ModelNames_Chosen =np.load(DATA_LOCATION+"Collect_ModelNames_Chosen.npy")
Collect_FeatIndies = np.load(DATA_LOCATION+"CLL_TIM_FeatIndexes.npy", allow_pickle=True)
ALLFeats = np.load(DATA_LOCATION+"ALLFeats_.npy")
#FuncFeatNames = pd.read_csv(DATA_LOCATION+"FeatureFunctionalNames.csv", sep="\t", header=None)
FuncFeatNames = pd.read_csv(DATA_LOCATION+"FeatureFunctionalNames_Danish.txt", sep="\t", header=None,encoding="ISO-8859-1")


FAllInds = 0
for nn in range(0, CurrEnsSize):
   FAllInds = np.append(FAllInds, Collect_FeatIndies.item()[Collect_CVData_Chosen[nn, 0]])
FAInds = np.unique(FAllInds)
################################################################################
# Setting Up Patient Feature Vector
################################################################################
CT_FNM = np.load(DATA_LOCATION+"CT_FNM.npy")
CT_FNM_Baseline = CT_FNM[CT_FNM[:, 1] == "Baseline", :]
CT_FNM_Labka = np.copy(LabbiesNames)
CT_FNM_BC = CT_FNM[CT_FNM[:, 2] == "BC", :]
# Generating Base-learner Predictions
#       models = [xgb.XGBClassifier(), xgb.RandomForestClassifier(), xgb.XGBClassifier(), xgb.XGBClassifier(), xgb.SGDClassifier(), xgb.XGBClassifier(), xgb.RandomForestClassifier(),
#               xgb.XGBClassifier(), xgb.ExtraTreesClassifier(), xgb.RandomForestClassifier(), xgb.XGBClassifier(), xgb.XGBClassifier(), xgb.XGBClassifier(), xgb.SGDClassifier(),
```

```
#                               xgb.RandomForestClassifier(), xgb.XGBClassifier(), xgb.LogisticRegression(), xgb.LogisticRegression(),
xgb.RandomForestClassifier(), xgb.XGBClassifier(), xgb.ExtraTreesClassifier(),
#                                   xgb.XGBClassifier(), xgb.ExtraTreesClassifier(), xgb.RandomForestClassifier(), xgb.XGBClassifier(),
xgb.XGBClassifier(), xgb.ExtraTreesClassifier(), xgb.RandomForestClassifier()]
base_learners = []
feats_of_bll_list = []
f_inds_list = []
for nn in range(0, CurrEnsSize):
    # Loading Base Learner
    if nn in indexXGB:
        Curr_BL = xgb.XGBClassifier()
        booster = xgb.Booster()
        booster.load_model(DATA_LOCATION+"CLL-TIM_BLN_" + str(nn) + ".sav-0" + ".bin") # SP Version
        #booster.load_model(DATA_LOCATION+"BL_NewSet/CLL-TIM_BLN_" + str(nn) + ".sav-0" + ".bin")

        Curr_BL._Booster = booster
        base_learners.append(Curr_BL)
    else:
        Curr_BL = pickle.load(open(DATA_LOCATION+"CLL-TIM_BLN_" + str(nn) + ".sav", "rb")) # SP Version
        #Curr_BL = pickle.load(open(DATA_LOCATION+"BL_NewSet/CLL-TIM_BLN_" + str(nn) + ".sav", "rb"))
        base_learners.append(Curr_BL)
    # Loading feature indices of base-learner
    FInds = Collect_FeatIndies.item()[Collect_CVData_Chosen[nn, 0]]
    fcnt = 0
    FeatsofBL = np.zeros((len(FInds))) - 99
    for ff in FInds:
        FeatsofBL[fcnt] = np.squeeze(np.argwhere(FAInds == ff))
        fcnt = fcnt + 1
    FeatsofBL = np.int64(FeatsofBL)
    feats_of_bll_list.append(FeatsofBL)
    f_inds_list.append(FInds)
All_CLL_TIM_Res = pd.DataFrame([])
All_OutputVal = pd.DataFrame([])
PAll_Data=PAll_Data.rename(columns={"PatientEpicId":"Patient",
                        "DiagnosisDate":"Diag_Date",
                        "Labanalysis":"Variable_Name",
                        "Value":"Value_of_Test",
                        "Unit":"Units_of_Test",
                        "CollectionInstant":"Date_of_Test"
                        })
PAll_Data["Variable_Category"]="lab"

# Bloodculture processing
i='H:\Rudi\CLL_TIM_SP_Monitoring/all_bloodcultures.xlsx'
passwd="jhds87y!hg"
decry_wb=io.BytesIO()
with open(i,'rb') as file:
    of=msoffcrypto.OfficeFile(file)
    of.load_key(password=passwd)
    of.decrypt(decry_wb)

all_bloodcultures=pd.read_excel(decry_wb)
all_bloodcultures['LabCollectionInstant']=pd.to_datetime(all_bloodcultures['LabCollectionInstant'])
all_bloodcultures=pd.merge(all_bloodcultures,PAll_Basic,left_on="PatientIdDurableKey",right_on="PatientEpicId")
all_bloodcultures['DaysToInfec']=(all_bloodcultures['LabCollectionInstant']-all_bloodcultures['Prediction_date']).dt.days
all_bloodcultures_features=all_bloodcultures[all_bloodcultures['DaysToInfec']<0]

all_bloodcultures_features['Variable_Name']='infec'
all_bloodcultures_features['Value_of_Test']=1
all_bloodcultures_features['Units_of_Test']=np.nan
all_bloodcultures_features['Variable_Category']='bc'

all_bloodcultures_features=all_bloodcultures_features.rename(columns={"PatientIdDurableKey":"Patient",
                        "DiagnosisDate":"Diag_Date",
                        "Prediction_date":"Prediction_date",
                        "LabCollectionInstant":"Date_of_Test",

                        })

all_bloodcultures_features.reset_index(inplace=True,drop=True)
all_bloodcultures_features_final=all_bloodcultures_features[['Patient', 'Diag_Date', 'Prediction_date', 'Variable_Name',
       'Value_of_Test', 'Units_of_Test', 'Date_of_Test', 'Variable_Category']]


PAll_Data["Date_of_Test"]=pd.to_datetime(PAll_Data["Date_of_Test"])
```

```
PAll_Data=pd.concat([PAll_Data,all_bloodcultures_features_final])
# End of Blood culture Processing


# Create variable to identify runs in the log
logpatientnum = 1
for patient_id in PAll_Basic.PatientEpicId:
    P01_Basic = (PAll_Basic[PAll_Basic["PatientEpicId"] == patient_id]).copy().reset_index(drop=True)
    P01_Data = (PAll_Data[PAll_Data["Patient"] == patient_id]).copy().reset_index(drop=True)

    #################################
    # SP to REDCAP format conversion
    #################################
    P01 = pd.DataFrame(P01_Data.copy().reset_index(drop=True))


    P01 = P01.append({"Variable_Category": "dm",
                      "Variable_Name": "sex",
                      "Value_of_Test": P01_Basic["Sex"][0],
                      }, ignore_index=True)
    P01 = P01.append({"Variable_Category": "dm",
                      "Variable_Name": "agereg",
                      "Value_of_Test": P01_Basic["Years"][0],
                      }, ignore_index=True)
    P01["Patient"] = P01_Basic["PatientEpicId"][0]
    P01["Diag_Date"] = P01_Basic["DiagnosisDate"][0]
    P01["Prediction_date"]= P01_Basic["Prediction_date"][0] ### ADDED




    for key in Units:
        P01["Units_of_Test"][P01["Units_of_Test"]==key]=Units[key]




    for key in LabelConv:
        P01["Variable_Name"][P01["Variable_Name"]==key]=LabelConv[key]



    # Binet Conversion
    cnt=1
    for cvec in np.array(["A","B","C","D"]):
        P01["Value_of_Test"][P01["Value_of_Test"]==("Stage "+cvec)]=cnt
        cnt=cnt+1

    cind=P01["Variable_Name"]=="binet"
    P01_binet=P01[cind].reset_index(drop=True)
    P01=P01[cind==False].reset_index(drop=True)
    P01_binet=P01_binet.sort_values("Date_of_Test").drop_duplicates(["Variable_Name"],keep="last")
    P01=pd.concat([P01,P01_binet]).reset_index(drop=True)
    P01["Variable_Category"][P01["Variable_Name"]=="binet"]="dm"


    # IGHV Conversion
    cind=P01["Variable_Name"]=="ighv"
    P01_ighv=P01[cind].reset_index(drop=True)
    P01=P01[cind==False].reset_index(drop=True)
    P01_ighv=P01_ighv.sort_values("Date_of_Test").drop_duplicates(["Variable_Name"],keep="last")
    P01=pd.concat([P01,P01_ighv]).reset_index(drop=True)
    P01["Variable_Category"][P01["Variable_Name"]=="ighv"]="dm"
    cind=P01["Variable_Name"]=="ighv"
    if np.logical_or(P01["Value_of_Test"][cind].values=="Positive",P01["Value_of_Test"][cind].values=="Muteret"):
        P01["Value_of_Test"][cind]="mu"
    elif  np.logical_or(P01["Value_of_Test"][cind].values=="Negative",P01["Value_of_Test"][cind].values=="Umuteret"):
        P01["Value_of_Test"][cind]="unmu"
    if P01["Value_of_Test"][cind].values=="Unknown":
        P01["Value_of_Test"][cind]="NA"
```

```
# Gender Conversion
cind=P01["Variable_Name"]=="sex"
if P01["Value_of_Test"][cind].values=="Mand":
    P01["Value_of_Test"][cind]=1
elif  P01["Value_of_Test"][cind].values=="Kvinde":
    P01["Value_of_Test"][cind]=2
P01["Variable_Category"][P01["Variable_Name"]=="sex"]="dm"
# CLL baseline markers
P01["Variable_Category"][P01["Variable_Name"]=="13q"]="dm"
P01["Variable_Category"][P01["Variable_Name"]=="17p"]="dm"
P01["Variable_Category"][P01["Variable_Name"]=="tri12"]="dm"
P01["Variable_Category"][P01["Variable_Name"]=="11p"]="dm"
P01["Variable_Category"][P01["Variable_Name"]=="cd38"]="dm"
P01["Variable_Category"][P01["Variable_Name"]=="zap70"]="dm"
P01["Variable_Category"][P01["Variable_Name"]=="ecog"]="dm"
P01["Variable_Category"][P01["Variable_Name"]=="famcll"]="dm"
############################
# Laboratory Data Cleaning (NOTE, need to add same day mean collapse)
############################
cind=P01["Variable_Category"]=="lab"
P01_lab=P01[cind].reset_index(drop=True)
P01=P01[cind==False].reset_index(drop=True)
DropInd=np.zeros((P01_lab.shape[0]))
for pp in range(P01_lab.shape[0]):
    try:
        P01_lab.Value_of_Test[pp]=float(P01_lab.Value_of_Test[pp])
    except:
        DropInd[pp]=1
P01_lab=P01_lab[DropInd==0].reset_index(drop=True)
P01=pd.concat([P01,P01_lab]).reset_index(drop=True)

ToRemo = np.squeeze(np.argwhere(np.logical_and(P01["Variable_Category"] == "lab",
pd.isnull(P01["Value_of_Test"])).values))
P01 = P01.drop(ToRemo).reset_index()
P01.loc[P01.Value_of_Test.isnull(),"Value_of_Test"]="NA"


###################################
# Date Format Conversion
#######################################
# Diagnosis Date
# correct dato format already provided in SQL ## DateSplit=P01.Diag_Date.str.split("/",expand=True)
# correct dato format already provided in SQL ## P01["Diag_Date"]=DateSplit[2]+"-"+DateSplit[1]+"-"+DateSplit[0]
# Date of Test
DateSplit=P01.Date_of_Test.astype(str).str.split(" ",expand=True)
P01["Date_of_Test"]=DateSplit[0].copy()

P01["Prediction_Point"]=todayF1
P01["Prediction_Point"]=P01.Prediction_date
P01["Diag_Date"]=pd.to_datetime(P01["Diag_Date"])
P01["Date_of_Test"]=pd.to_datetime(P01["Date_of_Test"])
P01["Prediction_Point"]=pd.to_datetime(P01["Prediction_Point"])
DiagDate=P01["Diag_Date"][0]
PPoint=P01["Prediction_Point"][0]

########      END OF SP TO REDCAP FORMAT CONVERSION       ############


################################################################################





# Feature Extraction
################################################################################


#################################################################################
# Extracting baseline variables and creating baseline features

Baseline_P = np.zeros((1, 32))
# Going through Each of the Baseline Features Features and checking if they are in Patient Data
```

```
bcnt = -1
for CurrBVar in CT_FNM_Baseline[:, 0]:
    CB = CurrBVar.split("_")[0]
    bcnt = bcnt + 1

    CurrBVar_T = RCBaselineList[np.argwhere(CB == np.array(BaselineList_Orig))[0][0]]  # translated baseline name
    ConVal = -99
    if np.sum(CurrBVar_T == P01["Variable_Name"].values) > 0:

        Where = np.argwhere(CurrBVar_T == P01["Variable_Name"].values)[0][0]

        if "binet" in CB:

            if "_A" in CurrBVar:
                if P01["Value_of_Test"][Where] == 1:
                    ConVal = 1
                else:
                    ConVal = 0
            if "_B" in CurrBVar:
                if P01["Value_of_Test"][Where] == 2:
                    ConVal = 1
                else:
                    ConVal = 0

            if "_C" in CurrBVar:
                if P01["Value_of_Test"][Where] == 3:
                    ConVal = 1
                else:
                    ConVal = 0


        elif "Age" in CB:
            ConVal = np.int64(P01["Value_of_Test"][Where])/100

        elif "WHOPERFORMANCE" in CB:

            if "Can Walk" in CurrBVar:
                if P01["Value_of_Test"][Where] == 1:
                    ConVal = 1
                else:
                    ConVal = 0
            if "Good Condition" in CurrBVar:
                if P01["Value_of_Test"][Where] == 0:
                    ConVal = 1
                else:
                    ConVal = 0


        elif "Gender" in CB:
            if P01["Value_of_Test"][Where] == 2: #female
                ConVal = 1
            elif P01["Value_of_Test"][Where] == 1: #male
                ConVal = 0
            else:
                ConVal = -99
        elif "umut" in CB:
            if "umut_1" in CurrBVar:

                if P01["Value_of_Test"][Where] == "unmu":
                    ConVal = 1
                else:
                    ConVal = 0
            if "umut_0" in CurrBVar:

                if P01["Value_of_Test"][Where] == "mu":
                    ConVal = 1
                else:
                    ConVal = 0
            if "umut_NA" in CurrBVar:

                if P01["Value_of_Test"][Where] == "NA":
                    ConVal = 1
                else:
                    ConVal = 0
```

```
        else: # includes del13,del11,tri12,del17


            if np.logical_or(P01["Value_of_Test"][Where] == 0,P01["Value_of_Test"][Where] == "Negative"):
                ConVal = 1
            else:
                ConVal = 0
            if "_1" in CurrBVar:

                if np.logical_or(P01["Value_of_Test"][Where] == 1,P01["Value_of_Test"][Where] == "Positive"):
                    ConVal = 1
                else:
                    ConVal = 0

            if "_NA" in CurrBVar:
                  if np.logical_or(P01["Value_of_Test"][Where]=="nd",P01["Value_of_Test"][Where]=="Not assessed"):
                        ConVal=1
                  else:
                        ConVal=0
            if "_NA" in CurrBVar:
                  if np.logical_or(P01["Value_of_Test"][Where]==99,P01["Value_of_Test"][Where]=="Ikke undersøgt"):
                        ConVal=1
                  else:
                        ConVal=0

    Baseline_P[0, bcnt] = ConVal


Baseline_P = np.squeeze(np.copy((Baseline_P)))
Baseline_P[Baseline_P == -99] = 0
if Baseline_P[2]==0:
    Baseline_P[3]=1
if np.logical_and(Baseline_P[4]==0,Baseline_P[5]==0):
    Baseline_P[6]=1
if np.logical_and(Baseline_P[7]==0,Baseline_P[8]==0):
    Baseline_P[9]=1
if np.logical_and(Baseline_P[10]==0,Baseline_P[11]==0):
    Baseline_P[12]=1
if np.logical_and(Baseline_P[15]==0,Baseline_P[16]==0):
    Baseline_P[17]=1

if np.logical_and(Baseline_P[21]==0,Baseline_P[22]==0):
    Baseline_P[23]=1
if np.logical_and(Baseline_P[24]==0,Baseline_P[25]==0):
    Baseline_P[26]=1







########## APPLYING UNIT CONVERSIONS ########################################

for tcnt in range(np.shape(P01)[0]):
    if P01.iloc[tcnt]["Variable_Category"]=="lab":
        CurrDaty=P01.iloc[tcnt]["Date_of_Test"]
        CurrVary=P01.iloc[tcnt]["Variable_Name"]
        CurrValue=float(P01.iloc[tcnt]["Value_of_Test"])
        CurrUnit=P01.iloc[tcnt]["Units_of_Test"]
        if CurrVary=="b2m":
            if CurrUnit=="mg_dL":
                P01.at[tcnt,"Value_of_Test"]=str(CurrValue*10) # to mg_L
            elif CurrUnit=="mmol_L":
                P01.at[tcnt,"Value_of_Test"]=str(CurrValue*1000000*0.0118) # to nmol_L to  mg_L
            elif CurrUnit=="nmol_L":
                P01.at[tcnt,"Value_of_Test"]=str(CurrValue*0.0118) #  to  mg_L


        if CurrVary=="crp":
            if CurrUnit=="nmol_L":
                P01.at[tcnt,"Value_of_Test"]=str(CurrValue/9.5238) # to mg_L
```

```
        if CurrVary=="hema":
            if CurrUnit=="percentage":
                P01.at[tcnt,"Value_of_Test"]=str(CurrValue/100) # fraction

        if CurrVary=="hbm":
            if CurrUnit=="mg_dL":
                P01.at[tcnt,"Value_of_Test"]=str((CurrValue/1000)*0.6206) # to g_dl to mmol_L
            if CurrUnit=="g_dL":
                P01.at[tcnt,"Value_of_Test"]=str(CurrValue*0.6206) #to g_dl to mmol_L

        if CurrVary=="trans":
            if CurrUnit=="g_L":
                P01.at[tcnt,"Value_of_Test"]=str(CurrValue*12.5628) # mcmol_L

        if CurrVary=="neutro":
            if CurrUnit=="percentage":
                wr=np.argwhere(np.logical_and(P01["Variable_Name"]=="wbc",P01["Date_of_Test"]==CurrDaty)==1)[0][0]
                CurrWBC=float(P01.iloc[wr]["Value_of_Test"])
                P01["Variable_Name"]=="wbc"
                P01.at[tcnt,"Value_of_Test"]=str(CurrWBC*(CurrValue/100)) # to fraction to  10^9/L

        if CurrVary=="baso":
            if CurrUnit=="percentage":
                wr=np.argwhere(np.logical_and(P01["Variable_Name"]=="wbc",P01["Date_of_Test"]==CurrDaty)==1)[0][0]
                CurrWBC=float(P01.iloc[wr]["Value_of_Test"])
                P01["Variable_Name"]=="wbc"
                P01.at[tcnt,"Value_of_Test"]=str(CurrWBC*(CurrValue/100)) # to fraction to 10^9/L

############################################################

# Adding beta2m at baseline
Entered=0
if np.sum(P01["Variable_Name"]=="b2m")>0:
    b2ms=0
    Where = "b2m" == P01["Variable_Name"]
    VChunk = np.squeeze(np.column_stack((P01["Date_of_Test"][Where], P01["Value_of_Test"][Where])).astype(float))
    VChunk=(P01[["Date_of_Test","Value_of_Test"]][Where]).reset_index(drop=True)

    if np.sum(Where)==1:
        if abs(VChunk.Date_of_Test[0]-DiagDate)<=timedelta(days=30):
            Entered=1
            b2ms=np.append(b2ms,VChunk.Value_of_Test[0])
    else:
        for n in range(np.sum(Where)):

            if abs(VChunk.Date_of_Test[n]-DiagDate)<=timedelta(days=30):
              Entered=1
              b2ms=np.append(b2ms,VChunk.Value_of_Test[n])

    if Entered==1:
        b2ms=np.nanmean(b2ms[1:])
        if b2ms<4:
            Baseline_P[18]=1
        else:
            Baseline_P[19]=1


if np.logical_and(Baseline_P[18]==0,Baseline_P[19]==0):
    Baseline_P[20]=1

CT_FNM_Baseline[Baseline_P==1]
################################################################################
################################################################################
### Extracting Lab Variables and Creating Lab Features
Lab_P = np.empty((1, 120))
Lab_P[:] = np.nan

VV = np.copy(CT_FNM_Labka)
VV[:,1] = np.where((VV[:,1]!=365) & (VV[:,1]!=90), 365*20, VV[:,1])



for v, CL in enumerate(CT_FNM_Labka[:, 0]):
```

```
CurrLVar_T = RCLabList[np.argwhere(CL == np.array(LabList_Orig))[0][0]]  # translated baseline name
ConVal = -99
if np.sum(CurrLVar_T == P01["Variable_Name"]) > 0:
    CurrFeatVal=np.nan
    ConVal = -99
    Where = CurrLVar_T == P01["Variable_Name"]




    VChunk=(P01[["Date_of_Test","Value_of_Test"]][Where]).reset_index(drop=True)


    ToKeep1=(PPoint-VChunk["Date_of_Test"])>=timedelta(days=0)
    ToKeep2=VChunk["Date_of_Test"] >= (PPoint - timedelta(days=VV[v, 1]))

    ToKeep = np.logical_and(ToKeep1, ToKeep2)
    VChunk = VChunk[ToKeep].reset_index(drop=True)
    if len(VChunk) != 0:



        CLDates = VChunk["Date_of_Test"]
        CLValues = np.abs(VChunk["Value_of_Test"].values.astype(float))

        Un_Dates_L = np.shape(CLDates)[0]

        CLDates= (PPoint-CLDates).dt.days.values

        if np.shape(VChunk)[0]>1:
              indios=np.argsort(CLDates)
              CLDates=CLDates[indios]
              CLValues=CLValues[indios]

        if VV[v, 2] == "Instance":
            if VV[v, 3] == "Cnt":
                CurrFeatVal=np.shape(VChunk)[0] # we cnt identical  test on same day as multiple in here
            elif VV[v, 3] == "latest":
                CurrFeatVal = np.min(CLDates)
            elif VV[v, 3] == "Mean":
                CurrFeatVal = np.nanmean(CLDates)
        elif VV[v, 2] == "nanmax":
            CurrFeatVal = np.nanmax(CLValues)
        elif VV[v, 2] == "nanmin":
            CurrFeatVal = np.nanmin(CLValues)
        elif VV[v, 2] == "nanmean":
            CurrFeatVal = np.nanmean(CLValues)
        elif VV[v, 2] == "nanmedian":
            CurrFeatVal = np.nanmedian(CLValues)
        elif VV[v,2]=="nanstd":
              if len(CLValues)!=1:
                    CurrFeatVal=np.nanstd(CLValues,ddof=1)
              else:
                    CurrFeatVal=0
        elif VV[v,2]=="kurtosis":
              if len(CLValues)!=1:
                    if len(np.unique(CLValues))==1:
                          CurrFeatVal=np.nan
                    else:
                          CurrFeatVal=scipy.stats.kurtosis(CLValues,axis=0, fisher=False)
              else:
                    CurrFeatVal=np.nan
        elif VV[v,2]=="skewness":

              if len(CLValues)!=1:
                    if len(np.unique(CLValues))==1:
                          CurrFeatVal=np.nan
                    else:
                          CurrFeatVal=scipy.stats.skew(CLValues)
              else:
                    CurrFeatVal=np.nan
        if  Un_Dates_L>=3:
              if VV[v,2]=="kurtosis":
                    if len(np.unique(CLValues))==1:
```

```
                    CurrFeatVal=np.nan
                else:
                    CurrFeatVal=scipy.stats.kurtosis(CLValues,axis=0, fisher=False)
            elif VV[v,2]=="skewness":
                if len(np.unique(CLValues))==1:
                    CurrFeatVal=np.nan
                else:
                    CurrFeatVal=scipy.stats.skew(CLValues)

            elif VV[v,2]=="Slope1":

                if VV[v,3]=="coefa":
                    Slp1=np.polyfit(CLDates,CLValues,1)
                    CurrFeatVal=Slp1[0]
                elif VV[v,3]=="coefb":
                    Slp1=np.polyfit(CLDates,CLValues,1)
                    CurrFeatVal=Slp1[1]

            elif VV[v,2]=="Slope2":
                if VV[v,3]=="coefa":

                    Slp1=np.polyfit(CLDates,CLValues,2)
                    CurrFeatVal=Slp1[0]
                elif VV[v,3]=="coefb":

                    Slp1=np.polyfit(CLDates,CLValues,2)
                    CurrFeatVal=Slp1[1]
                elif VV[v,3]=="coefc":

                    Slp1=np.polyfit(CLDates,CLValues,2)
                    CurrFeatVal=Slp1[2]

        if Un_Dates_L>=2:
            if VV[v,2]=="kurtosis":
                if len(np.unique(CLValues))==1:
                    CurrFeatVal=np.nan
                else:
                    CurrFeatVal=scipy.stats.kurtosis(CLValues,axis=0, fisher=False)
            elif VV[v,2]=="skewness":
                if len(np.unique(CLValues))==1:
                    CurrFeatVal=np.nan
                else:
                    CurrFeatVal=scipy.stats.skew(CLValues)


        Lab_P[0,v]=CurrFeatVal



Lab_P = np.squeeze(np.copy(np.transpose(Lab_P)))
################################################################################

################################################################################
# Extracting Infection Dates and Applying Infection Features

InfecDates = np.unique(P01["Date_of_Test"]["infec" == P01["Variable_Name"]])
InfecDates=pd.to_datetime(InfecDates)


if InfecDates.shape[0]!=0:
    print(InfecDates)

    #Dates to Integers
    I_PPoint=0
    I_InfecDates=-(PPoint - InfecDates).days


    ToKeep1 = (I_PPoint - I_InfecDates) > 0 # No Future Points
    Infec_90 = I_PPoint - I_InfecDates[np.logical_and(I_InfecDates >= (I_PPoint - 90), ToKeep1)]
    Infec_90=Infec_90.values
    Infec_365 = I_PPoint - I_InfecDates[np.logical_and(I_InfecDates >= (I_PPoint - 365), ToKeep1)]
    Infec_365=Infec_365.values
    Infec_Inf = I_PPoint - I_InfecDates[np.logical_and(I_InfecDates >= (I_PPoint - 365 * 20), ToKeep1)]
    Infec_Inf=Infec_Inf.values
```

```
    Infec_90 = np.int64(Infec_90.astype(None))
    SInfec_90 = Infec_90[np.argsort(-Infec_90)]
    Infec_365 = np.int64(Infec_365.astype(None))
    SInfec_365 = Infec_365[np.argsort(-Infec_365)]
    Infec_Inf = np.int64(Infec_Inf.astype(None))
    SInfec_Inf = Infec_Inf[np.argsort(-Infec_Inf)]
else:
    ToKeep1=np.array([])
    Infec_90=np.array([])
    Infec_365=np.array([])
    Infec_Inf=np.array([])
    SInfec_90=np.array([])
    SInfec_365=np.array([])
    SInfec_Inf=np.array([])

# if no infections = zero
IFeats = np.zeros((12, 1))

if len(SInfec_Inf)>=3:
        Slp=np.polyfit(np.arange(1,len(SInfec_Inf)+1),SInfec_Inf,1)
        IFeats[0]=Slp[0] # slope1_coefa

if len(SInfec_90)>=3:
        Slp=np.polyfit(np.arange(1,len(SInfec_90)+1),SInfec_90,2)
        IFeats[1]=Slp[2] # slope2_coefc

if len(Infec_90>0):
        IFeats[2]=np.nanmean(Infec_90)
        IFeats[5]=np.nanmin(Infec_90)
        if len(Infec_Inf)!=1:
              if len(np.unique(CLValues))==1:
                    IFeats[7]=np.nan
              else:
                    IFeats[7]=scipy.stats.skew(Infec_90)
        else:
              IFeats[7]=np.nan
if len(Infec_365)>0:
        IFeats[3]=np.nanmean(Infec_365)
        if len(Infec_365)!=1:
              IFeats[4]=np.nanstd(Infec_365,ddof=1)
        else:
              IFeats[4]=0
if len(Infec_Inf)>0:
        IFeats[6]=np.nanmax(Infec_Inf)
        if len(Infec_Inf)!=1:
              if len(np.unique(Infec_Inf))==1:
                    IFeats[8]=np.nan
              else:
                    IFeats[8]=scipy.stats.kurtosis(Infec_Inf,axis=0, fisher=False)
        else:
              IFeats[8]=np.nan

if len(SInfec_Inf)==2:
        Changes=SInfec_Inf[0]-SInfec_Inf[1]
        IFeats[9]=np.nanmin(Changes)
        IFeats[11]=np.nanmean(Changes)
elif len(SInfec_Inf)>2:

        Changes=SInfec_Inf[0:-1]-SInfec_Inf[1:];
        IFeats[9]=np.nanmin(Changes)
        IFeats[11]=np.nanmean(Changes)

if len(SInfec_365)==2:
        Changes=SInfec_365[0]-SInfec_365[1]
        IFeats[10]=np.nanmean(Changes)
elif len(SInfec_365)>2:

        Changes=SInfec_365[0:-1]-SInfec_365[1:];
        IFeats[10]=np.nanmean(Changes)

if len(Infec_90)==0:
        IFeats[1]=0
        IFeats[2]=0
        IFeats[5]=0
        IFeats[7]=0
```

```
elif len(Infec_90)==1:
        IFeats[7]=np.nan
        IFeats[1]=np.nan
elif len(Infec_90)==2:
        IFeats[1]=np.nan

if len(Infec_365)==0:
        IFeats[3]=0
        IFeats[4]=0
        IFeats[10]=0

elif len(Infec_365)==1:
        IFeats[10]=np.nan

if len(Infec_Inf)==0:
        IFeats[0]=0
        IFeats[6]=0
        IFeats[8]=0
        IFeats[9]=0
        IFeats[11]=0
elif len(Infec_Inf)==1:
        IFeats[8]=np.nan
        IFeats[0]=np.nan
        IFeats[9]=np.nan
        IFeats[11]=np.nan
elif len(Infec_Inf)==2:
        IFeats[0]=np.nan

#print(IFeats)
Bw90=0
Bw365=0
BwInf=0

IFeats = np.squeeze(IFeats)
Infec_P = np.copy(np.transpose(IFeats))

OutputVal = np.concatenate((Baseline_P, np.zeros((64,)), Lab_P, Infec_P))  # Joining Feature Vectors (
OutputVal[32]=Bw90
OutputVal[33]=Bw365
OutputVal[34]=BwInf

# Turns Binet Missing into Stage A
Bin=OutputVal[27] + OutputVal[28] + OutputVal[29]
if Bin==0:
    OutputVal[27]=1


CheckFeats = np.column_stack((CT_FNM, OutputVal))
# END OF FEATURE GENERATION #

################################################################################
# Generating 28 Base-learner Predictions
################################################################################
AllProbs = np.zeros((28, 1))
FinalFeats = np.zeros((1, 228))
RealFeats = np.zeros((1, 228))
RealFeatsU = np.zeros((1, 228))
PopMatrix = np.zeros((4149, 228))
PopMatrixU = np.zeros((4149, 228))
FinalFeatsMat =np.zeros((28,228))-999
VMatrix_orig = np.row_stack((OutputVal, OutputVal))


for nn in range(0, CurrEnsSize):
    Curr_BL = base_learners[nn]
    FeatsofBL = feats_of_bll_list[nn]
    FInds = f_inds_list[nn]


    AATrain = TrainMatrix[:, FInds]


    VMatrixU= VMatrix_orig[:, FeatsofBL]
    VMatrix = VMatrix_orig[:, FeatsofBL]
```

```
    if Collect_ModelNames_Chosen[nn, 1] != "XGB":  # normalization if not XGBoost

        [TMatrix, get_colmean] = Nan2Mean(TrainMatrix[:, FInds])
        VMatrix = Nan2Mean_useColmean(VMatrix, get_colmean)
        scaler = preprocessing.StandardScaler()
        scaler.fit(TMatrix)
        TMatrix = scaler.transform(TMatrix)
        VMatrix = scaler.transform(VMatrix)

    AATrainNorm = TrainMatrix[:, FInds]

    mx = np.nanmax(AATrainNorm, axis=0)
    mn = np.nanmin(AATrainNorm, axis=0)
    AATrainNorm = (AATrainNorm - mn) / (mx - mn)
    VMatrixNorm = (VMatrix - mn) / (mx - mn)
    Probs = Curr_BL.predict_proba(VMatrix)

    AllProbs[nn] = Probs[0, 1]
    if (Collect_ModelNames_Chosen[nn, 1] == np.array(["Elastic", "LogisticRegression"])).any():



        X_train_summary = shap.kmeans(TMatrix, 10)
        explainer = shap.LinearExplainer(Curr_BL, VMatrix)


        shap_values = explainer.shap_values(VMatrix)

    elif (Collect_ModelNames_Chosen[nn, 1] == np.array(["XGB"])).any():
        explainer = shap.TreeExplainer(Curr_BL)
        shap_values = explainer.shap_values(VMatrix)
    elif (Collect_ModelNames_Chosen[nn, 1] == np.array(["RF","ExtraTreesClassifier"])).any():
        explainer = shap.TreeExplainer(Curr_BL)
        shap_values = explainer.shap_values(VMatrix)
        shap_values = shap_values[0]
    cnty = 0
    for s in TransList[FInds]:
        cind = np.int64(np.squeeze(np.argwhere(ALLFeats == s)))

        if cind.size > 0:


            FinalFeats[0, cind] = FinalFeats[0, cind] + shap_values[0, cnty]
            RealFeats[0, cind] = VMatrixNorm[0, cnty]
            RealFeatsU[0, cind] = VMatrixU[0,cnty]
            PopMatrixU[:, cind] = AATrain[:, cnty]
            PopMatrix[:, cind] = AATrainNorm[:, cnty]
            cnty = cnty + 1



ProbRisk = np.mean(AllProbs)  # Generating Mean Probabilistic Risk from CLL-TIM
# END OF CLL-TIM Prediction
################################################################################

################################################################################
## Generation of Personalized Risk Factors
################################################################################
Toppies = ["", "", "", "", "", "", "", "", "", ""]
ToppiesContribs = np.zeros((1, 10))
ToppiesRealVals = np.zeros((1, 10))
ToppiesRealValsU = np.zeros((1, 10))
TopInds = np.zeros((1, 10))
for n in range(np.shape(FinalFeats)[0]):

    Top = np.argsort(-1 * FinalFeats[n, :])
    TopVals = np.sort(-1 * FinalFeats[n, :])

    TopLR = np.array(FuncFeatNames[0][Top[0:5]])
    for rr in range(5):
        if FuncFeatNames[2][Top[rr]] == 1:
            if RealFeatsU[0, Top[rr]] == 0:
                TopLR[rr] = FuncFeatNames[1][Top[rr]]
        else:
```

```
                if np.isnan(RealFeatsU[0, Top[rr]]):
                    TopLR[rr] = FuncFeatNames[1][Top[rr]]

        TopHR = np.array(FuncFeatNames[0][Top[-5:]])


        rrcnt = -1
        for rr in np.array([223, 224, 225, 226, 227]):
            rrcnt = rrcnt + 1
            if FuncFeatNames[2][Top[rr]] == 1:
                if RealFeatsU[0, Top[rr]] == 0:
                    TopHR[rrcnt] = FuncFeatNames[1][Top[rr]]
            else:

                if np.isnan(RealFeatsU[0, Top[rr]]):
                    TopHR[rrcnt] = FuncFeatNames[1][Top[rr]]

        Toppies = np.row_stack((Toppies, np.append(TopHR, TopLR)))
        ToppiesContribs = np.row_stack((ToppiesContribs, np.append(TopVals[-5:], TopVals[0:5])))
        ToppiesRealVals = np.row_stack((ToppiesRealVals, np.append(RealFeats[n, Top[-5:]], RealFeats[n, Top[0:5]])))
        ToppiesRealValsU = np.row_stack((ToppiesRealValsU, np.append(RealFeatsU[n, Top[-5:]], RealFeatsU[n, Top[0:5]])))
        TopInds = np.row_stack((TopInds, np.append(Top[-5:], Top[0:5])))

    ToppiesRealVals = ToppiesRealVals[1:, :]
    ToppiesRealValsU = ToppiesRealValsU[1:, :]
    ToppiesContribs = ToppiesContribs[1:, :]
    Toppies = Toppies[1:, :]
    TopInds = np.int64(TopInds[1:, :])
    ################################################################################



    PRFOut_HR=""
    PCnt=0
    #if ProbRisk > 0.5:
    for nnn in range(5, 10):
        PCnt = PCnt + 1
        if np.logical_or(FuncFeatNames[3][TopInds[0, nnn]] == "B", np.isnan(RealFeatsU[0, TopInds[0, nnn]])):
            PRFOut_HR=np.append(PRFOut_HR,str(PCnt) + ": " + Toppies[0, nnn])
        else:



            if ToppiesRealValsU[0, nnn] <= np.nanmedian(PopMatrixU[:, TopInds[0, nnn]]):
                PRFOut_HR=np.append(PRFOut_HR,str(PCnt) + ": " + Toppies[0, nnn] + " Med en værdi på " + str(
                    ToppiesRealValsU[0, nnn]) + " lavere end befolkningsmedianen på  " +
                      str(np.round(np.nanmedian(PopMatrixU[:, TopInds[0, nnn]]), 1)))
            else:


                PRFOut_HR=np.append(PRFOut_HR,str(PCnt) + ": " + Toppies[0, nnn] + " Med en værdi " + str(
                    ToppiesRealValsU[0, nnn]) + " højere end befolkningsmedianen på " +
                      str(np.round(np.nanmedian(PopMatrixU[:, TopInds[0, nnn]]), 1)))
#else:
#if ProbRisk > 0.5:
    PRFOut_LR=""
    PCnt=0
    for nnn in range(4, -1, -1):
        PCnt = PCnt + 1
        if np.logical_or(FuncFeatNames[3][TopInds[0, nnn]] == "B", np.isnan(RealFeats[0, TopInds[0, nnn]])):


            PRFOut_LR=np.append(PRFOut_LR,str(PCnt) + ": " + Toppies[0, nnn])


        else:

            if ToppiesRealValsU[0, nnn] <= np.nanmedian(PopMatrixU[:, TopInds[0, nnn]]):

                PRFOut_LR=np.append(PRFOut_LR,str(PCnt) + ": " + Toppies[0, nnn] + " Med en værdi på " + str(
                    ToppiesRealValsU[0, nnn]) + " lavere end befolkningsmedianen på  " +
                      str(np.round(np.nanmedian(PopMatrixU[:, TopInds[0, nnn]]), 1)))
            else:

                PRFOut_LR=np.append(PRFOut_LR,str(PCnt) + ": " + Toppies[0, nnn] + " Med en værdi " + str(
```

```
                ToppiesRealValsU[0, nnn]) + " højere end befolkningsmedianen på " +


                 str(np.round(np.nanmedian(PopMatrixU[:, TopInds[0, nnn]]), 1)))
    ################################################
    # Gathering CLL-TIM Output
    ################################################
    if ProbRisk > 0.5:
        Risk="høj"
        if ProbRisk > 0.58:
            Conf="høj"
        else:
            Conf="lav"
    else:
        Risk="lav"
        if ProbRisk < 0.28:
            Conf="høj"
        else:
            Conf="lav"

    Risk_Level=ProbRisk
    Risk_Std=np.std(AllProbs)

    CLL_TIM_Res={"PID":patient_id,
                "Forudsigelsesdag": PPoint,
                "Risiko":Risk,
                "Konfidens":Conf,
                "Risikoniveau":Risk_Level,
                "Risikostandardafvigel":Risk_Std,
                "Højrisikofaktor 1":str(PRFOut_HR[1]),
                "Højrisikofaktor 2":str(PRFOut_HR[2]),
                "Højrisikofaktor 3":str(PRFOut_HR[3]),
                "Højrisikofaktor 4":str(PRFOut_HR[4]),
                "Højrisikofaktor 5":str(PRFOut_HR[5]),
                "Lavrsikofaktor 1":str(PRFOut_LR[1]),
                "Lavrsikofaktor 2":str(PRFOut_LR[2]),
                "Lavrsikofaktor 3":str(PRFOut_LR[3]),
                "Lavrsikofaktor 4":str(PRFOut_LR[4]),
                "Lavrsikofaktor 5":str(PRFOut_LR[5]),
                }

    CLL_TIM_Res=pd.DataFrame(data=CLL_TIM_Res,index=[0])
    All_CLL_TIM_Res=pd.concat([All_CLL_TIM_Res,CLL_TIM_Res]).reset_index(drop=True) # Used for Metric Calculation
    OutputVal=pd.DataFrame(data=OutputVal)
    All_OutputVal=pd.concat([All_OutputVal,OutputVal],axis=1).reset_index(drop=True) # Used for Metric Calculation

    logpatientnum = logpatientnum + 1


data_out = pd.DataFrame({"Risiko": All_CLL_TIM_Res["Risiko"],
                        "PatientEpicId": All_CLL_TIM_Res["PID"],
                        "Konfidens": All_CLL_TIM_Res["Konfidens"],
                        "Højrisikofaktor_1": All_CLL_TIM_Res["Højrisikofaktor 1"],
                        "Højrisikofaktor_2": All_CLL_TIM_Res["Højrisikofaktor 2"],
                        "Højrisikofaktor_3": All_CLL_TIM_Res["Højrisikofaktor 3"],
                        "Højrisikofaktor_4": All_CLL_TIM_Res["Højrisikofaktor 4"],
                        "Højrisikofaktor_5": All_CLL_TIM_Res["Højrisikofaktor 5"],
                        "Lavrsikofaktor_1": All_CLL_TIM_Res["Lavrsikofaktor 1"],
                        "Lavrsikofaktor_2": All_CLL_TIM_Res["Lavrsikofaktor 2"],
                        "Lavrsikofaktor_3": All_CLL_TIM_Res["Lavrsikofaktor 3"],
                        "Lavrsikofaktor_4": All_CLL_TIM_Res["Lavrsikofaktor 4"],
                        "Lavrsikofaktor_5": All_CLL_TIM_Res["Lavrsikofaktor 5"],
                        "Risikoniveau": All_CLL_TIM_Res["Risikoniveau"],
                        "Risikostandardafvigel": All_CLL_TIM_Res["Risikostandardafvigel"]})
```


1. Feng J, Phillips RV, Malenica I, et al. Clinical artificial intelligence quality improvement: towards continual monitoring and updating of AI algorithms in healthcare. *npj Digital Med*. 2022;5(1):66. doi:10.1038/s41746-022-00611-y

2. Da Cunha-Bang C, Agius R, Kater AP, et al. PreVent-ACaLL Short-term combined acalabrutinib and venetoclax treatment of newly diagnosed patients with CLL at high risk of infection and/or early treatment, who do not fulfil IWCLL treatment criteria for treatment. A randomized study with extensive immune phenotyping. *Blood*. 2019;134(Supplement_1):4304-4304. doi:10.1182/blood-2019-121907

3. Agius R, Brieghel C, Andersen MA, et al. Machine learning can identify newly diagnosed patients with CLL at high risk of infection. *Nat Commun*. 2020;11(1):363. doi:10.1038/s41467-019-14225-8
